# The health and economic burden of musculoskeletal disorders in Belgium from 2013 to 2018

**DOI:** 10.1186/s12963-023-00303-z

**Published:** 2023-04-21

**Authors:** Vanessa Gorasso, Johan Van der Heyden, Robby De Pauw, Ingrid Pelgrims, Eva M. De Clercq, Karin De Ridder, Stefanie Vandevijvere, Stijn Vansteelandt, Bert Vaes, Delphine De Smedt, Brecht Devleesschauwer

**Affiliations:** 1 Department of Epidemiology and Public Health, Sciensano, Rue J Wytsman 14, 1050 Brussels, Belgium; 2 Department of Public Health and Primary Care, Ghent University, Ghent, Belgium; 3 Department of Rehabilitation Sciences, Ghent University, Ghent, Belgium; 4 Department of Chemical and Physical Health Risks, Sciensano, Brussels, Belgium; 5 Department of Applied Mathematics, Computer Science and Statistics, Ghent University, Ghent, Belgium; 6 Department of Medical Statistics, London School of Hygiene and Tropical Medicine, London, UK; 7 Department of Public Health and Primary Care, KU Leuven, Leuven, Belgium; 8 Department of Translational Physiology, Infectiology and Public Health, Ghent University, Merelbeke, Belgium

**Keywords:** Musculoskeletal disorders, Disability-adjusted life years, Healthcare costs, Absenteeism costs

## Abstract

**Introduction:**

Low back pain (LBP), neck pain (NKP), osteoarthritis (OST) and rheumatoid arthritis (RHE) are among the musculoskeletal (MSK) disorders causing the greatest disability in terms of Years Lived with Disability. The current study aims to analyze the health and economic impact of these MSK disorders in Belgium, providing a summary of morbidity and mortality outcomes from 2013 to 2018, as well as direct and indirect costs from 2013 to 2017.

**Methods:**

The health burden of LBP, NKP, OST and RHE in Belgium from 2013 to 2018 was summarized in terms of prevalence and disability-adjusted life years (DALY) using data from the Belgian health interview surveys (BHIS), the INTEGO database (Belgian registration network for general practitioners) and the Global Burden of Diseases study 2019. The economic burden included estimates of direct medical costs and indirect costs, measured by cost of work absenteeism. For this purpose, data of the respondents to the BHIS-2013 were linked with the national health insurance data (intermutualistic agency [IMA] database) 2013–2017.

**Results:**

In 2018, 2.5 million Belgians were affected by at least one MSK disorder. OST represented the disorder with the highest number of cases for both men and women, followed by LBP. In the same year, MSK disorders contributed to a total of 180,746 DALYs for female and 116,063 DALYs for men. LBP appeared to be the largest contributor to the health burden of MSK. Having at least one MSK disorder costed on average 3 billion € in medical expenses and 2 billion € in indirect costs per year, with LBP being the most costly.

**Conclusion:**

MSK disorders represent a major health and economic burden in Belgium. As their burden will probably continue to increase in the future, acting on the risk factors associated to these disorders is crucial to mitigate both the health and economic burden.

**Supplementary Information:**

The online version contains supplementary material available at 10.1186/s12963-023-00303-z.

## Background

According to world health organization (WHO), approximately one in three people worldwide live with a chronic, painful musculoskeletal (MSK) disorder. Within the European Union, MSK disorders represent the most prevalent and most costly work-related health problems affecting about 45 million workers [1]. In addition to workplace conditions, lack of physical activity and obesity are major risk factors for many MSK disorders [2]. Often disregarded due to their low fatality rate, MSK conditions account for the greatest proportion of persistent pain, affecting physical function and mental health, increasing risk of developing other chronic health conditions and all-cause mortality [3, 4]. Low back pain (LBP), neck pain (NKP), osteoarthritis (OST) and rheumatoid arthritis (RHE) are the MSK disorders with the highest impact in terms of years lived with disability (YLD). Moreover, according to the Global Burden of Disease (GBD) study of 2019, LBP is the leading global cause of YLDs [5]. OST showed an increase in prevalence and is predicted to be one of the leading future causes of burden of disease [6, 7].

Although the prevalence of most of these conditions increases with age, middle-aged people during their peak income-earning years are predominantly affected by LBP and NKP [8]. MSK health is therefore essential for maintaining economic, social and functional independence, as well as human capital across the life course. In high-income countries, MSK disorders are the major contributors to the loss of productive life years in the workforce compared with other non-communicable diseases (NCD), commonly resulting in early retirement and reduced financial security [9]. *Likewise,* MSK disorders contribute sizeably to direct health expenses. In 2006, the Belgian Federal Healthcare Knowledge Centre (KCE) estimated the direct cost of LBP in Belgium to be 272 million euros [10]. More recent estimates, as well as estimates for other MSK disorders, are however lacking.

An up-to-date and complete summary of the burden of the most important MSK disorders can inform health authorities on the urgent matter and be used as a base for the prioritization of the interventions. This study aims to analyze the health and economic impact of the most common MSK disorders (LBP, NKP, OST, RHE) in Belgium, providing a summary of morbidity and mortality outcomes, as well as cost estimates.

## Methods

Both the health and the economic burden of LBP, NKP, OST and RHE in Belgium were estimated. The health burden was summarized in terms of prevalent cases and prevalence rates for the years 2013–2018 by sex and age group. Mortality estimates were considered only for RHE, following the below-described GBD methodology, from 2013 to 2018. Disability-Adjusted Life Years (DALY) were used to summarize the impact of the concerned diseases both in terms of mortality and morbidity. The economic burden included the direct and indirect health costs attributable to the selected MSK disorders for the years from 2013 to 2017 expressed in mean yearly attributable cost and total yearly cost.

### Prevalence and mortality

Prevalence estimates for LBP, NKP and OST were derived from the Belgian health interview surveys (BHIS) conducted in 2013 and 2018 among a representative sample of the Belgian population (*N* = 10,829 participants in 2013 and *N* = 11,611 participants in 2018). Respondents were 15 years or older and were recruited following a multistage sampling design. The sampling and recruitment procedure are described in Demarest et al. [11]. Interviews were performed using a face-to-face paper and pencil interview supplemented with a self-administered questionnaire covering more sensitive topics [11]. The data included self-reported information on health status and related health behavior and determinants. Socio-demographic information such as age, gender, household educational level (i.e., the highest educational level within the household), income level (based on the calculated quintiles of the household income) and behavioral risk factors were included from the BHIS database. To identify persons suffering from chronic diseases, respondents were asked the following; “Did you suffer from ‘*disease of interest*’ in the past 12 months?” In addition, LBP and NKP cases were assigned only if respondents also reported a very bad to fair subjective health. This was done to avoid overestimation since LBP and NKP are not necessarily chronic conditions, differently from OST and RHE. Prevalence was computed by gender, 5 years age group and region (i.e., Brussels, Flanders and Wallonia). Considering that the BHIS does not provide information on cases for the < 15 years old category, we assumed the prevalence to be zero. Prevalence estimates for the years between 2013 and 2018 were calculated by means of linear interpolation based on the estimates derived from BHIS-2013 and BHIS-2018.

Lack of specificity (many false positives) in the RHE data was suspected looking at the high prevalence rates from the BHIS [12]. Therefore, a more accurate data source was used for the prevalence estimates of RHE. They were derived from the Intego network, which represents a registration network for general practices in Flanders (Belgium). It includes anonymized diagnoses, laboratory results and drug prescriptions from 55 general practitioners (GPs) [13, 14]. RHE cases were identified through International Classification of Primary Care (ICPC) code L88. Data were delivered by 10-year age groups and sex from year 2013 to 2018. In order to obtain prevalence proportions, numbers of cases were divided by the yearly contact group (i.e., the number of different patients seen by the 55 GPs), available from the Intego database. Since these estimates are available for only one of the three Belgian regions, national estimates were inferred in the following way: (1) compute the proportion of cases in the other regions compared to Flanders using RHE estimates within BHIS, i.e., prevalence of RHE in Brussels/Wallonia divided by prevalence of RHE in Flanders for 2013 and 2018; (2) multiply the proportions derived in (1) by the prevalence of RHE in Flanders observed within Intego for obtaining the new prevalence of RHE cases in Brussels and Wallonia. The prevalence estimates in Brussels and Wallonia between 2013 and 2018 were computed by means of linear interpolation as done for the other MSK disorders.

Number of deaths between 2013 and 2018 were retrieved from Statbel, the Belgian statistical office, for people with RHE (ICD-10 codes: M05, M06, M08) as underlying cause of death. This included also the deaths attributed to RHE during the redistribution process of ill-defined causes of death in Belgian BoD study. Following the GBD methodology, LBP, NKP and OST were assumed to not generate any deaths [15].

### Disability-adjusted life years

DALYs are calculated by summing the Years Lived with Disability (YLD), representing the morbidity component and the Years of Life Lost (YLL), the mortality component. In order to compute the YLDs, the number of prevalent cases (prevalence times population) derived from BHIS were multiplied by the disability weights (DW) retrieved from the GBD-2019, using the disease models of the GBD study [15]. Population information was extracted by 5-year age groups and sex from Statbel for the years from 2013 to 2018. For RHE, the YLLs were calculated subtracting the individual age at death from the GBD-2019 standard life expectancy [16]. We report the 100,000 DALY rates and age-standardized rates, using Belgian 2018 population as reference.

In order to account for the overlap among these disorders, we used a multiplicative approach to compute comorbidity-adjusted YLD. The prevalence estimates of reporting one, two, or three of this conditions were extrapolated from BHIS for LBP, NKP and OST. Secondly DW were adjusted using the following formula 1 − ∏_*i*_(1 − DW_*i*_), with DW_*i*_ being the disease-specific DW [17, 18]. Considering that LBP, NKP and OST were extrapolated from the same data source, these were considered as interdependent based on the observed correlations. For RHE, we assumed that it was independent of the other health conditions.

### Costs

This analysis included the estimation of (1) direct costs, measured by reimbursed expenditures for medical services and medications associated with treatment and care; and (2) indirect costs, measured by cost of work absenteeism.

Data of the respondents to the BHIS-2013 were linked with national health insurance data compiled by the Intermutualistic Agency (IMA) 2013–2017. Health insurance is compulsory in Belgium covering more than 99% of the population. Linkage is performed via the national registry number at individual level. In a later stage household composition according to HIS and IMA information is compared and, based on date of birth, sex and date of the interview, the national register number of the other household members is retrieved. However, if HIS respondents are reported as household member by the reference person, but this deviates from the “official” situation in the national register, no linkage was possible.

The IMA database comprised reimbursed total healthcare costs, for every payment modality (directly paid by the health insurance, patients out-of-pocket and supplements). These expenditures included (1) ambulatory care (over-the-counter pharmaceuticals excluded), (2) hospital care and (3) reimbursed medicines purchased through pharmacies. Available information on hospital care only included variable costs. However, in Belgium, the national health insurance also pays a fixed amount to the hospitals per admitted patient, depending on the type of hospital and treatment. Precise information on these costs was not directly available in the dataset. In order to estimate the fixed part of the total hospital care cost, the hospitalizations per patient per year were multiplied with the average annual 100% per diem cost publicly available by type of hospitalization (per diem costs available through: https://www.riziv.fgov.be/nl/themas/kost-terugbetaling/door-ziekenfonds/verzorging-ziekenhuizen/Paginas/verpleegdagprijzen-ziekenhuizen.aspx).

Costs of absenteeism were estimated based on the question available in the BHIS-2013: “Have you been absent from work during the past 12 months due to health problems? In doing so, take into account any conditions, injuries or other health problems you may have had and which resulted in an absence from work.” Followed by the question: “How many days in total have you been absent from work for the past 12 months due to health problems? If you are unable to indicate this number of days correctly, please give an estimate.” However, since respondents might have included weekend days in their answer, the maximum number of working days per year, i.e., 226 days, was considered. If the answer given exceeded this number, the maximum number of working days was used. The productivity loss was valued using the human capital approach, assuming that each day of absence corresponds to the average gross daily wage [19]. The number of days absent from work was then multiplied by the national yearly average labour cost per hour multiplied by an average of 7.6 h (38 h per week) per working day [20, 21]. The labour cost includes compensation for employees as well taxes without subsidies. The individual cost of absenteeism over the 2013–2017 period was obtained by multiplying the annual individual cost for the days absent from work based on the days reported in the BHIS-2013 and the national yearly average labour cost of the corresponding years. Only the working population was included in the calculation (respondents who stated having a paid job at the time of the survey).

**Fig. 1:**
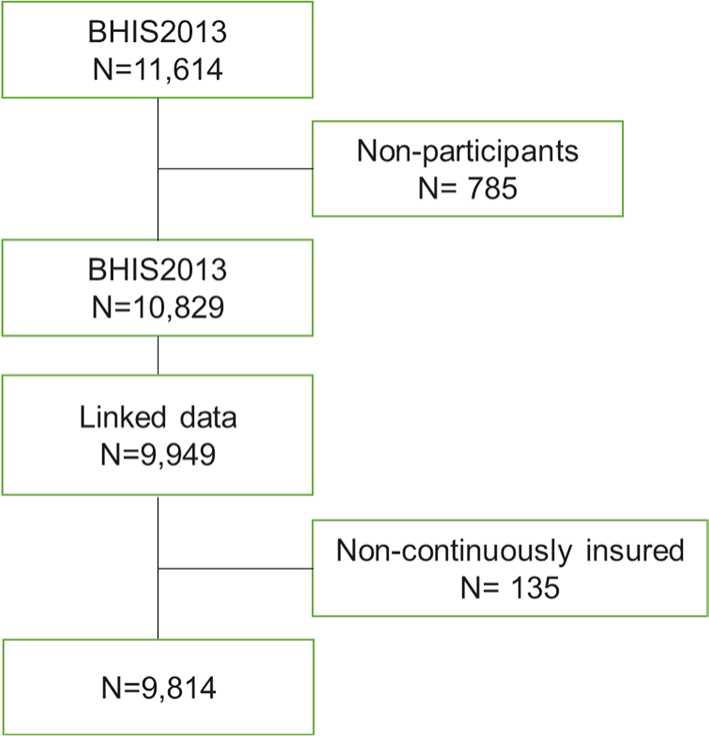
Sample selection process for the healthcare costs. After excluding non-participants from the BHIS2013 dataset, we merged it with IMA dataset, resulting in 9949 participants. Additionally, people for whom the health insurance organization did not have continuous data were excluded

Figure 1 shows the sample selection process, including data merging, used for the computation of cost estimates between 2013 and 2017. People who were not continuously insured throughout the observed time period (2013–2017) were excluded. People who died within the observational period were excluded for their direct and indirect costs in the years after their death. The final sample resulted in 9814 respondents.

### Statistical analyses

Prevalence and mortality estimates were presented as number of cases and as percentages of cases over the total population per age group, sex and year. In order to have an estimate for the overall Belgian population, the survey design (weights, strata and cluster) of the BHIS-2013 and BHIS-2018 was duly taken into account. The detailed computation of the weights is described elsewhere [11, 22]. DALYs adjusted for the co-occurrence of the considered MSK conditions were summed up by age group and sex. Confidence intervals (95% CI) were calculated for prevalence estimates based on the standard errors resulting from the survey analysis. We assumed a beta distribution to sample the DW and the proportion of people in each health states using the mean and confidence intervals derived from GBD 2019 literature [15]. Uncertainty was propagated to DALYs using 1000 Monte Carlo simulations.

Incremental costs were estimated at the individual level using the method of recycled predictions, also known as G-computation or direct standardization, that allows to estimate the marginal effect of each MSK disorder on healthcare costs [23, 24]. The latter was computed with a multistep approach: (1) the coefficients of the regression model (described above) were used to predict healthcare costs for each respondent using the observed distribution of the concerned MSK disorders as reported in BHIS-2013; (2) the same coefficients were then used to predict healthcare cost assuming that none of the respondents had the concerned MSK disorder, keeping all other characteristics as observed (including the status of the other MSK disorders); (3) the difference of the above-described predictions represents the individual incremental cost of the concerned MSK disorder; (4) finally, we calculated the attributable cost of LBP and NKP as the survey weighted average of these individual incremental costs. Univariate and multivariable regressions with negative binomial distribution and log link were used to explore the extent to which average yearly cost was associated with MSK disorders. The first was used to estimate an unadjusted incremental cost with only the disease as independent variable. Multivariable regressions additionally included socio-demographic characteristics and lifestyle factors: age-groups, gender, household educational level, mean household income level, high body mass index (BMI), smoking, alcohol misuse, unhealthy eating behavior (i.e., drinking sugared soft drinks daily) and physical inactivity. A “double-selection” approach was used for the selection of the variables to be included in the final multivariable model [25], i.e., the variables were identified by the means of two regression models, finding those that predict the dependent variable (average costs) and those that predict the independent variables (each MSK disorder separately). The final linear regression included all MSK disorders and all the covariates significant in the models above-described after a backwards elimination at a 10% level of acceptance. In addition to covariate adjustments, the multivariable regressions all included mediation analysis for estimating the direct cost of OST and RHE. Considering that LBP and NKP are also symptoms for OST and RHE and that the cost attributable to OST and RHE might be partially mediated through LBP and NKP, the above calculated costs represented only the costs directly attributable to the considered disorder, and ignoring other disorders. In order to calculate the total costs attributable to OST and RHE, inspired by causal mediation analyses [26], we first used the disease models of LBP and NKP (LBP/NKP as dependent variable, OST, RHE and covariates as independent variables) to stochastically impute the disease status of LBP and NKP in the absence of OST and RHE. We next applied the above-described four steps, where in step 2 we set LBP and NKP to these imputed disease statuses. Because the latter were stochastically imputed, this Monte Carlo simulation process was repeated 1000 times and results were averaged across simulations.

95% CIs were computed with a bootstrap approach with 1000 replicates. Total disease costs were computed by multiplying the previously calculated survey weighted average cost for the disease by the prevalence of the disease. Total costs for all MSK disorders were estimated by multiplying the mean per capita incremental cost by the prevalence of at least one MSK disorder in the general Belgian population for the direct costs and in the working Belgian population according to BHIS-2018.

All analyses were performed in R version 1.4.1717 [27]; the survey weighted analyses were performed using the survey package [28].

## Results

### Prevalence and mortality

Prevalence estimates of the most common MSK disorders in Belgium showed that more women were affected than men (see Additional file 1: Appendix 1–8). Within the analyzed MSK disorders, OST represented the disorder with the highest number of cases for both men and women, followed by LBP. In 2018, the number of female cases was almost twice the number of NKP cases in men (8.6% vs 4.3%; 491,317 vs 238,121) and OST (18.8% vs 11.7%; 1,080,969 vs 652,997). The sex-related differences in LBP (11.4% vs 9.0%; 653,496 vs 501,717) and RHE (0.8% vs 0.5%; 47,828 vs 28,768) were less clear. All the included MSK disorders presented a peak of cases from the age 40–45 to 80–85 years. In general, all age groups and sexes showed an increase in the number of cases in the observed time span (2013–2018), confirmed by the rates per 100,000 (see Additional file 1: Appendix 1–8). Some exceptions could be seen for OST cases for the age categories 55–64 and 75–89.

Women died more frequently due to RHE compared to men. Nevertheless, both sexes had a similar distribution of deaths within the age categories. The highest number of deaths were registered in patients from 65 till 95 years old, whereas almost no death occurred before the age of 50.

### Disability-adjusted life years

**Fig. 2:**
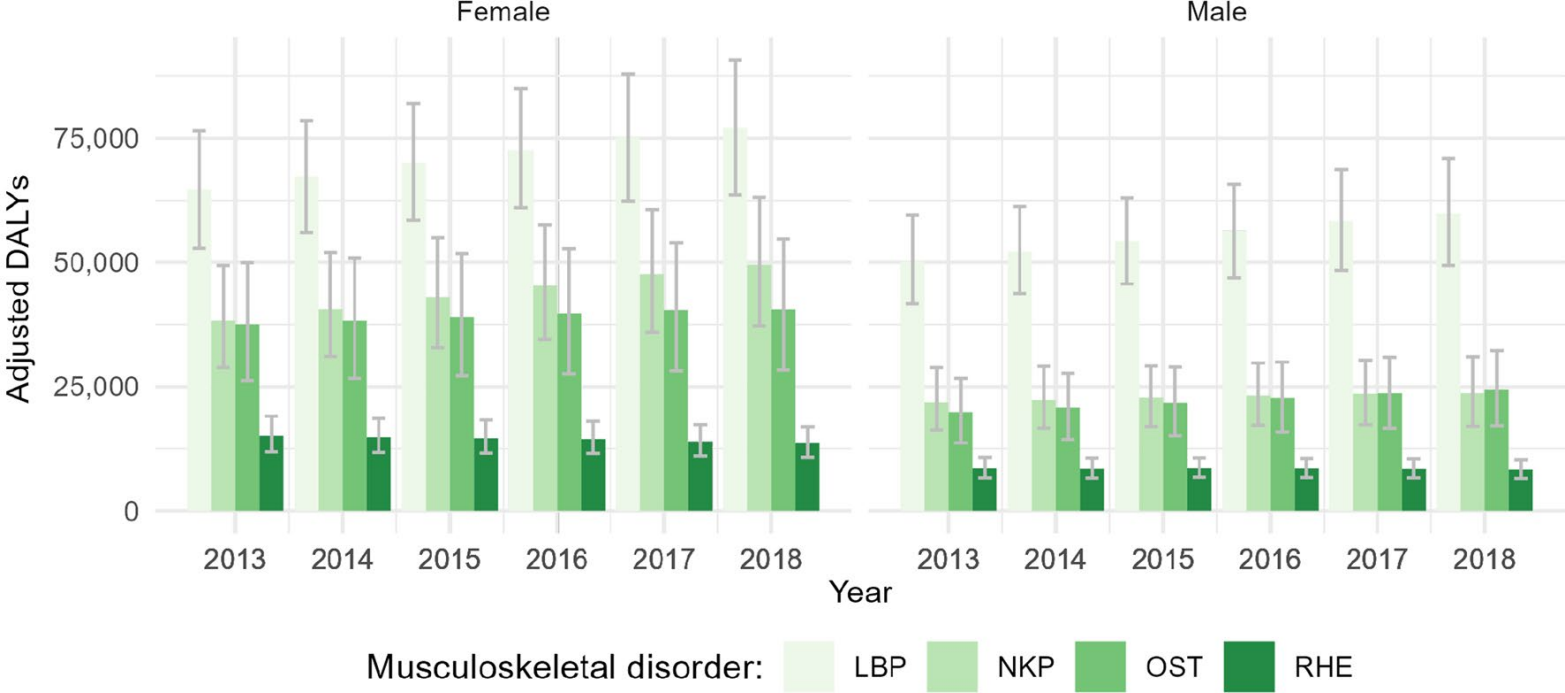
Adjusted disability-adjusted life years (adjusted for the co-occurrence of the other musculoskeletal disorders) and confidence intervals for the years 2013–2018 by musculoskeletal disorder and gender. [Disability-adjusted life years (DALY), low back pain (LBP), neck pain (NKP), osteoarthritis (OST), rheumatoid arthritis (RHE)]

As depicted in Fig. 2, LBP appeared to be the largest contributor to the health burden of MSK disorders for both genders. In 2018, a total of 137,323 DALY were estimated for LBP [female: 77,370 (95% CI 63,931; 91,192); male: 59,953 (95% CI 49,432; 71,055)], followed by 73,428 DALY for NKP [female: 49,731 (95% CI 37,363; 63,419); male: 23,697 (95% CI 17,022; 31,111)], 64,942 DALY for OST [female: 40,670 (95% CI 28,333; 54,750); male: 24,272 (95% CI 17,038; 32,156)] and 21,891 DALY for RHE [female: 13,580 (95% CI 1076; 16,894); male: 8239 (95% CI 6482; 10,280)]—see Additional file 1: Appendix 1–8. In RHE patients, YLD represented the greatest part of DALY. Notably, in 2018 Belgian women experienced 848 YLL and 12,733 YLD, as opposed to 238 YLL and 8001 YLD in men.

**Fig. 3:**
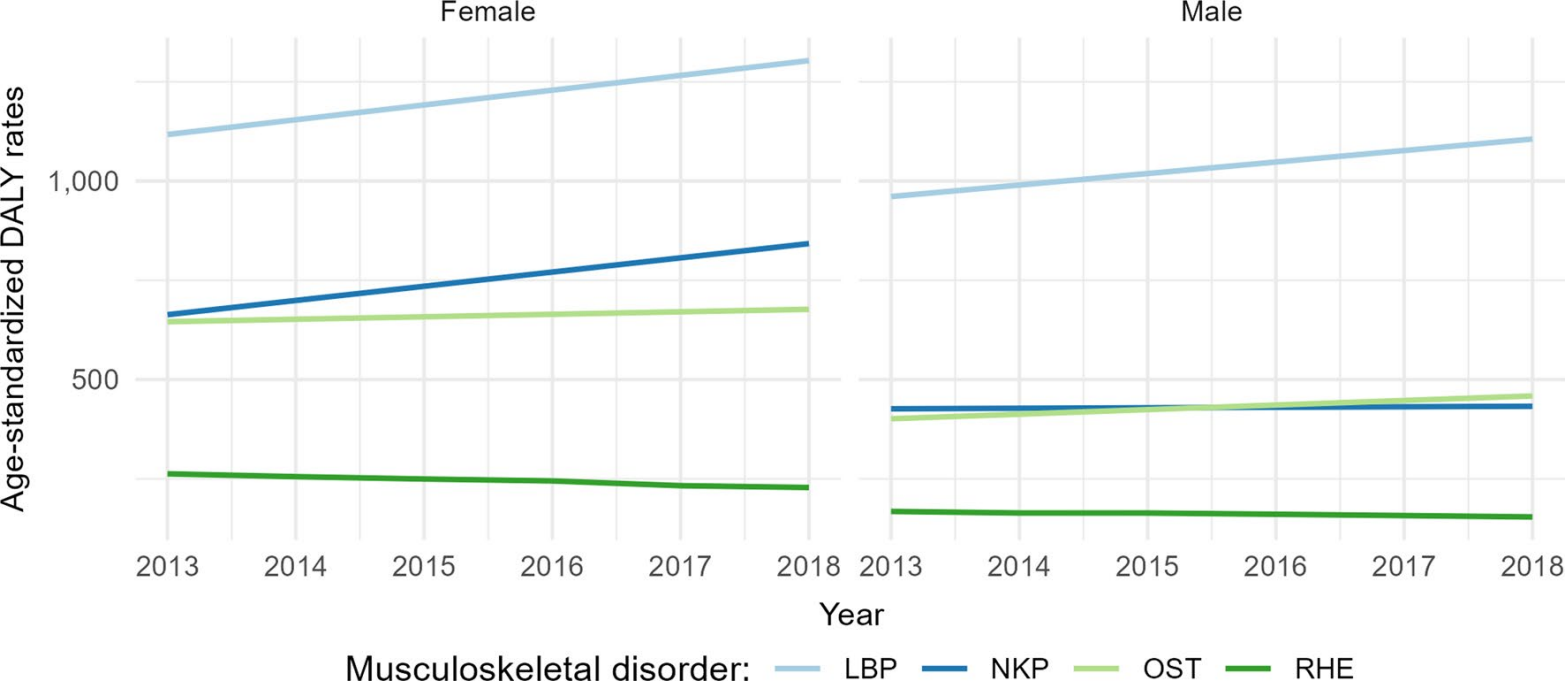
Age-standardized disability-adjusted life years 100,000 rate for the years 2013–2018 by musculoskeletal disorder and gender. [Disability-adjusted life years (DALY), low back pain (LBP), neck pain (NKP), osteoarthritis (OST), rheumatoid arthritis (RHE)]

Over the years, the female population had an increasingly higher burden of disease attributable to MSK disorders, as shown in Fig. 3 (see also Additional file 1: Appendix 1–8). In the male population, an increase in burden was also observed for LBP, NKP and OST. Moreover, RHE in both sexes was the only disorder to show a decrease throughout the observed period. The burden of NKP in females had the greatest increase from 2013 to 2018 compared to the other disorders (i.e., 26%).

### Direct costs attributable to musculoskeletal disorders

Additional file 1: Appendix 9 shows the sociodemographic characteristics of the selected sample and the survey-weighted percentages. The sample is representative for the adult Belgian population.

Results of the unadjusted model for direct costs attributable to MSK disorders are presented in Additional file 1: Appendix 10. These models were adjusted for confounding and mediation effect. The model included all four MSK disorders as well as sex, age, lack of physical activity, education level, BMI status and daily use of tobacco (resulted significant in the double selection process). The adjusted mean incremental cost showed that LBP is the MSK disorder with the highest direct cost [2405€ (95% CI 817€; 4069€) corresponding to 43% higher direct costs compared to a person without LBP], followed by NKP [2212€ (95% CI 275€; 4419€) corresponding to 36% higher direct costs compared to a person without LBP]. The cost ratios of OST (5%) and RHE (7%) were not statistically significant. Mean attributable costs also included the costs mediated through LBP and NKP, leading to a significant cost for OST [299€ (95% CI 23€; 733€)] but not for RHE [298€ (95% CI − 31€; 789€)] (Table 1).

**Table 1:** Adjusted direct annual costs ratios and mean incremental annual cost in function of musculoskeletal disorders (multivariable regression, Belgian adult population, from 2013 to 2017; *N* = 4591)

	Cost ratio (95% CI)	Mean attributable cost (95% CI)
MSK disorder (Ref: No)		
Low back pain	1.43** (1.15; 1.79)	2405€ (817€; 4069€)
Neck pain	1.36* (1.03; 1.8)	2212€ (275€; 4419€)
Osteoarthritis	1.05 (0.87; 1.27)	299€ (23€; 733€)
Rheumatoid arthritis	1.07 (0.86; 1.32)	298€ (− 31€; 789€)
Sex		
Male	Ref	
Female	0.83* (0.7; 0.97)	
Age	1.03*** (1.02; 1.03)	
Education		
Higher education	Ref	
Higher secondary	0.97 (0.81; 1.15)	
Lower secondary	1.15 (0.92; 1.43)	
No diploma or primary education	1.49* (1.1; 2.02)	
At risk due to a lack of leisure time physical activity		
No	Ref	
Yes	1.44*** (1.23; 1.7)	
Heavy daily smokers		
No	Ref	
Yes	1.22 (0.92; 1.63)	
BMI status		
Underweight	1.20 (0.91; 1.57)	
Normal weight	Ref	
Overweight	1.03 (0.87; 1.23)	
Obese	1.00 (0.81; 1.25)	

***p < 0.0001, **p < 0.05, *p < 0.10, ‘ ’ *p* > 0.10; included covariates were significant in the two models of the double selection process as presented in the methods section. Intercept: 975.33€ ***(95% CI 547.93€; 1736.14€). Cost ratios are the exponential of the coefficients resulting from the univariate analysis. They should be interpreted as: increasing by one level the independent covariates multiplies the mean attributable costs by the cost ratio

### Indirect costs attributable to MSK disorders

For the analysis of the attributable indirect costs, only the employed population (3941 out of 9998 in 2013) was included. Additional file 1: Appendix 9 shows that sociodemographic characteristics slightly changed from the previous sample, with a higher percentage of individuals having a higher education and higher income.

Unadjusted results for indirect costs can be found in Additional file 1: Appendix 11. The results of the model were adjusted by all four MSK disorder as well as sex, age, lack of physical activity, BMI status and daily use of tobacco (resulted significant in the double selection process). Table 2 shows the adjusted mean yearly indirect cost for each MSK disorder as well as their cost ratios. People affected by LBP were observed to have 63% higher indirect costs than people without LBP, corresponding to 6002€ (95% CI 900€; 10,893€). The other MSK disorders did not show significantly higher indirect costs compared to a population without MSK disorders: 869€ (95% CI − 281€; 2287€) for OST and 962€ (95% CI − 687€; 3347€) for RHE.

**Table 2:** Adjusted indirect annual costs ratios and mean incremental annual cost in function of musculoskeletal disorders (multivariable regression, Belgian adult population, from 2013 to 2017; *N* = 2523)

	Cost ratio (95% CI)	Mean attributable cost (95% CI)
MSK disorder (Ref: No)		
Low back pain	2.63** (1.29; 5.35)	6002€ (900€; 10,893€)
Neck pain	0.96 (0.41; 2.24)	− 340€ (− 8694€; 7035€)
Osteoarthritis	1.43 (0.88; 2.32)	869€ (− 281€; 2287€)
Rheumatoid arthritis	1.33 (0.70; 2.53)	962€ (− 687€; 3347€)
Sex		
Male	Ref	
Female	1.81*** (1.30; 2.51)	
Age	1.00 (0.98; 1.01)	
At risk due to a lack of leisure time physical activity		
No	Ref	
Yes	1.14 (0.75; 1.75)	
Heavy daily smokers		
No	Ref	
Yes	1.35 (0.82; 2.21)	
BMI status		
Underweight	1.25 (0.48; 3.29)	
Normal weight	Ref	
Overweight	1.20 (0.87; 1.66)	
Obese	1.71 (0.88; 3.33)	

***p < 0.0001, **p < 0.05, *p < 0.10, ‘ ’ *p* > 0.10; included covariates that were significant in the two models of the double selection process as presented in the methods section. Intercept: 1806€ ***(95% CI 974€; 3352€)

### Total costs for MSK disorders

The average direct cost for the combination of the four MSK disorders amounted to 1519€ (95% CI 762€; 2419€). This led to a total direct cost of 3,025,775,962€ in Belgium in 2018. Absenteeism costs amounted to 3247€ (95% CI 1871€; 5174€), average cost for the combination of the included MSK disorders. In 2018 in Belgium, 2,029,791,516€ were lost due to work absenteeism due to at least one MSK disorder.

## Discussion

This study provides a summary of the impact that the most common MSK disorders have on the health and economic disease burden in Belgium. Results showed that the prevalence of the included disorders increased from 2013 to 2018. In 2018, 2.5 million Belgians were affected by at least one MSK disorder. The burden was higher in women and the gender disparities increased with age. This impact translated in a total of 180,746 DALYs for female and 116,063 DALYs for men in 2018. MSK disorders were found to have a substantial economic burden both in terms of direct expenses and in absenteeism costs. In total, having at least one MSK disorder costed yearly €3 billion in medical expenses and €2 billion in indirect costs, with LBP being the most costly.

When comparing health outcomes, OST was the most prevalent MSK disorder but it was also one of the least impactful in terms of DALYs and direct and indirect mean incremental cost compared to the other disorders. On the other hand, the prevalence of NKP was lower compared to OST and LBP but it turned out to be the most costly disorder, both in terms of direct and indirect costs.

Comparisons with GBD and other international studies confirmed our results in terms of DALY regarding the differences in sexes and the distribution among the age groups found within our study [15, 29]. In addition, prevalence percentages of our study are not far from the results reported in GBD 2019 study estimates for 2018. Our estimates of OST (15.4% vs 14.8%), RHE (0.7% vs 0.4%) and NKP (6.5% vs 4.0%) are higher than for GBD; whereas for LBP we estimated a lower percentage (10.2% vs 13%) [15].

All over Europe, MSK disorders have been recognized and addressed in the years as being the most common work-related health problem. In 2015, a European report showed that 62% of Belgian working population suffered from one or more MSK disorders in the previous 12 months [30], leading to a considerable societal impact in terms of productivity. It was also reported that almost one third of the European workers were affected by one or more MSK disorders and were not be able to continue working up to the age of 60 [30].

In Belgium the situation is not different. According to our estimates, every year more than €2 billion is lost due to work absenteeism caused by MSK disorders, corresponding to 0.3% of Belgian GDP in 2018. In Germany, for example, MSK and connective tissue disorders accounted for 17.2€ billion in loss of production (production loss costs based on labour costs) in 2016, and 30.4€ billion in loss of gross value added (loss of labour productivity). This represents 0.5% and 1.0% of Germany’s GDP, respectively [30]. We also showed that LBP is considered to have the highest total annual direct and indirect cost, with 3269€ and 6557€, respectively, average direct and indirect cost. A Swedish study showed a mean total cost of 2753€ per LBP episode in 2011 (of which 917€ direct and 1836€ indirect costs) [31].

The constant aging of the population will accentuate the burden of MSK disorders, nevertheless acting on certain lifestyle factors associated with MSK disorders can help mitigating both the health and economic burden. Actions to contrast the increasing burden of these conditions started in the European countries and targeted the work environment. Occupational Health and Safety practitioners all over the world have created workplace-based interventions to reduce the MSK burden based on their training, knowledge and experiences. Among these, stretching exercise programmes, vibration feedback on mouse use and workstation forearm supports had a positive effect in preventing upper extremity MSK disorders [32]. In the European Union, the need to tackle work-related MSK disorders by improving working environments and equipment has been addressed by encouraging and promoting these actions in the European countries [30]. Nevertheless, there are still important challenges in the implementation of workplace-based interventions. Poorly implemented interventions could not be expected to lead to sustainable change [32]. Along with occupational risk factors, other types of contributors to the attributable burden could be tackled. Prevention of obesity and physical inactivity are considered to be key primary prevention strategies for OST [33] and exercise alone or in combination with education is effective for preventing LBP [34]. According to our study, there are still gaps for improvement in the health system since a significant proportion of the Belgian population continues to live with disabling MSK conditions, irrespective of age and gender.

### Strengths and limitations

This study represents a summary of the health and economic burden of MSK disorders in Belgium using national data. Our study provides valuable information of the health burden that corresponds to a large part of the total burden of disease but is often disregarded due to low fatality rate. Our approach of recycled predictions has allowed us to compare direct and indirect healthcare costs while adjusting for confounding by including important sociodemographic and health status covariates in the models.

We acknowledge some limitations within our study. First of all, there are some limitations that are intrinsic of the nature of our data sources. Self-reported data, deriving from national surveys, are subject to recalling and social desirability biases. In addition, our national survey is subject to misclassification bias since respondents may not have sufficient medical knowledge to make the distinction between a degenerative joint disease (OST) and an inflammatory joint disease (RHE). Nevertheless, surveys represent an essential source of information for personal data, like sociodemographic characteristics, and chronic diseases that remain frequently undiagnosed so they are difficult to grasp with other types of data sources (e.g., LBP and NKP). The definition of LBP and NKP was adjusted with the question on subjective health to be able to exclude any asymptomatic case. We acknowledge that this might have resulted in some overestimation, because it could not be ascertained whether the poor self-reported health was related to the condition of interest. Moreover, we tried to use the best possible available data source for each chronic disease. For instance, in the case of RHE, we believed that Intego would provide more precise estimates than BHIS. Nevertheless, this might have affected comparability of estimates among the different MSK disorders and assumes that all patients with RHE are in care by, or visited, a GP. National claims data collected at population-level does not include services that are not covered by insurance (e.g., ambulant psychotherapy, limited reimbursements for physiotherapy). Even so, administrative data are an essential source for investigating the financial burden of healthcare and, in Belgium, IMA has relatively complete data on healthcare services in the hospital and ambulatory care. We observed that almost 10% of the BHIS sample could not be linked with IMA. Nevertheless, it should also be noticed that the linkage is not subject to any “consent bias” since participants were not asked for their consent to the linkage. In our uncertainty analysis, we assumed a beta distribution for the proportion of cases in each health state and for the corresponding DW, using the mean and uncertainty intervals derived from GBD 2019 literature. This differs from the GBD methods that compute uncertainty intervals using non-parametric methods. Some limitations might also be attributed to the correctness of the fitted models as well as possible failure to control sufficiently for confounding. We have tried to overcome the latter by increasing the chance of detecting measured confounders via the double-selection process, but we cannot exclude the possibility that certain relevant confounders were not available. Further investigation would also need to address multimorbidity as affecting the total health burden as well as health expenditure.

## Conclusion

Based on national health and financial estimates, we found that MSK disorders had a substantial health and economic impact in Belgium. Since 2013, the disability burden of LBP, NKP, OST and RHE has been increasing for both female and male, reaching a total of 180,746 DALY for female and 116,063 DALY for men in 2018. We also estimated that €3 and €2 billion are spent yearly to cover, respectively, the direct and indirect expenditures attributable to MSK disorders. Policies and interventions are urgently needed to reduce the prevalence of these disorders. In particular, actions could be taken to reduce the risk factors that contribute to this burden.

## Supplementary Information

**Additional file 1: Appendix Table 1.** Number of LBP cases and confidence intervals by sex, age and year. **Appendix Table 2.** YLDs of LBP and confidence intervals by sex, age and year. **Appendix Table 3.** NKP cases and confidence intervals by sex, age and year. **Appendix Table 4.** YLDs of NKP by sex, age and year. **Appendix Table 5.** Number of OST cases by sex, age and year. **Appendix Table 6.** YLDs for OST per sex, age and year. **Appendix Table 7.** Number of RHE cases by sex, age and year. **Appendix Table 8.** YLD for RHE and confidence intervals by sex, age and year. **Appendix Table 9.** Sociodemographic characteristics of participants to HIS 2013 - at the time of the survey. **Appendix Table 10.** Unadjusted direct average annual costs in function of MSK disorders (univariate regression, Belgian adult population). **Appendix Table 11.** Unadjusted indirect average annual costs in function of MSK disorders (univariate regression, Belgian working population). **Appendix Table 12.** Results of the double selection process (coefficients and standard errors). The cost models include the variables that were significant either in the disease or the cost model, as described in the methods section.

## Data Availability

All data generated or analyzed during this study are included in this published article and its Additional files.

## Abbreviations

BHIS: Belgian health interview survey
BMI: Body mass index
CI: Confidence intervals
DALY: Disability-adjusted life years
DW: Disability weight
GBD: Global burden of disease
GP: General practitioners
ICPC: International Classification of Primary Care
IMA: Intermutualistic agency
KCE: Belgian Federal Healthcare Knowledge Centre
LBP: Low back pain
MSK: Musculoskeletal
NCD: Non-communicable diseases
NKP: Neck pain
OST: Osteoarthritis
RHE: Rheumatoid arthritis
WHO: World Health Organization
YLD: Years lived with disability
