# Supplementary material for: The health and economic burden of musculoskeletal disorders in Belgium from 2013 to 2018
Source: Popul Health Metr. 2023 Apr 21;21:4. doi: 10.1186/s12963-023-00303-z (PMC10122398; doi:10.1186/s12963-023-00303-z)
Supplement: Supplementary file 1 — Additional file 1: Appendix Table 1. Number of LBP cases and confidence intervals by sex, age and year. Appendix Table 2. YLDs of LBP and confidence intervals by sex, age and year. Appendix Table 3. NKP cases and confidence intervals by sex, age and year. Appendix Table 4. YLDs of NKP by sex, age and year. Appendix Table 5. Number of OST cases by sex, age and year. Appendix Table 6. YLDs for OST per sex, age and year. Appendix Table 7. Number of RHE cases by sex, age and year. Appendix Table 8. YLD for RHE and confidence intervals by sex, age and year. Appendix Table 9. Sociodemographic characteristics of participants to HIS 2013 - at the time of the survey. Appendix Table 10. Unadjusted direct average annual costs in function of MSK disorders (univariate regression, Belgian adult population). Appendix Table 11. Unadjusted indirect average annual costs in function of MSK disorders (univariate regression, Belgian working population). Appendix Table 12. Results of the double selection process (coefficients and standard errors). The cost models include the variables that were significant either in the disease or the cost model, as described in the methods section. [file 12963_2023_303_MOESM1_ESM.docx]

**Supplementary file**

Appendix Table 1: Number of LBP cases and confidence intervals by sex, age and year

|  |  | **2013** | | | **2014** | | | **2015** | | | **2016** | | | **2017** | | | **2018** | | |
| --- | --- | --- | --- | --- | --- | --- | --- | --- | --- | --- | --- | --- | --- | --- | --- | --- | --- | --- | --- |
| **Sex** | **Age** | **N** | **2.5CI** | **97.5CI** | **N** | **2.5CI** | **97.5CI** | **N** | **2.5CI** | **97.5CI** | **N** | **2.5CI** | **97.5CI** | **N** | **2.5CI** | **97.5CI** | **N** | **2.5CI** | **97.5CI** |
| **F** | **<1** | - | - | - | - | - | - | - | - | - | - | - | - | - | - | - | - | - | - |
| **F** | **1-4** | - | - | - | - | - | - | - | - | - | - | - | - | - | - | - | - | - | - |
| **F** | **5-9** | - | - | - | - | - | - | - | - | - | - | - | - | - | - | - | - | - | - |
| **F** | **10-14** | - | - | - | - | - | - | - | - | - | - | - | - | - | - | - | - | - | - |
| **F** | **15-19** | 10,049 | 4,336 | 17,944 | 10,753 | 5,938 | 17,209 | 11,516 | 6,976 | 16,922 | 12,262 | 7,482 | 18,062 | 12,952 | 7,429 | 19,676 | 13,549 | 7,191 | 21,987 |
| **F** | **20-24** | 11,303 | 5,134 | 20,827 | 12,078 | 6,830 | 19,927 | 12,769 | 7,748 | 18,960 | 13,413 | 8,302 | 19,640 | 14,056 | 8,325 | 21,335 | 14,615 | 7,819 | 23,334 |
| **F** | **25-29** | 21,471 | 12,205 | 32,474 | 20,580 | 12,686 | 29,418 | 19,732 | 13,380 | 26,973 | 18,855 | 13,106 | 25,188 | 17,865 | 12,268 | 24,117 | 16,547 | 10,328 | 23,953 |
| **F** | **30-34** | 22,322 | 12,870 | 35,060 | 21,143 | 13,482 | 31,368 | 19,901 | 13,422 | 28,033 | 18,664 | 12,902 | 25,255 | 17,518 | 11,970 | 24,745 | 16,288 | 10,381 | 24,288 |
| **F** | **35-39** | 31,792 | 21,900 | 42,992 | 33,526 | 25,619 | 43,335 | 35,514 | 27,266 | 45,233 | 37,532 | 27,744 | 49,150 | 39,332 | 27,876 | 53,172 | 40,461 | 26,322 | 57,814 |
| **F** | **40-44** | 33,871 | 23,815 | 45,482 | 35,148 | 26,467 | 44,826 | 36,251 | 27,798 | 45,851 | 37,274 | 27,636 | 49,378 | 38,440 | 27,017 | 53,484 | 39,369 | 25,928 | 57,431 |
| **F** | **45-49** | 40,984 | 31,192 | 50,802 | 45,187 | 36,806 | 53,629 | 49,371 | 41,603 | 57,057 | 53,753 | 45,086 | 62,456 | 58,221 | 47,686 | 69,299 | 61,871 | 49,064 | 75,102 |
| **F** | **50-54** | 40,681 | 31,787 | 50,230 | 45,926 | 38,044 | 54,274 | 51,095 | 43,304 | 59,515 | 55,959 | 46,877 | 65,678 | 60,470 | 49,548 | 72,312 | 64,084 | 51,108 | 78,464 |
| **F** | **55-59** | 50,438 | 39,876 | 62,532 | 54,299 | 45,185 | 64,270 | 58,150 | 48,995 | 67,458 | 61,967 | 52,480 | 72,007 | 65,820 | 55,069 | 77,613 | 69,090 | 55,369 | 83,555 |
| **F** | **60-64** | 45,367 | 35,025 | 56,163 | 48,402 | 39,669 | 57,314 | 51,812 | 43,584 | 59,551 | 55,500 | 47,481 | 63,327 | 59,306 | 49,876 | 68,614 | 62,627 | 50,874 | 74,492 |
| **F** | **65-69** | 54,941 | 42,208 | 70,267 | 56,017 | 45,223 | 69,339 | 56,755 | 47,423 | 68,017 | 56,623 | 47,816 | 66,177 | 55,815 | 46,670 | 66,167 | 54,508 | 43,896 | 66,595 |
| **F** | **70-74** | 42,437 | 32,410 | 53,961 | 41,809 | 33,360 | 51,419 | 41,886 | 34,719 | 50,341 | 43,597 | 36,737 | 51,648 | 46,156 | 38,657 | 55,001 | 47,315 | 37,774 | 58,050 |
| **F** | **75-79** | 52,519 | 43,019 | 63,160 | 53,380 | 45,437 | 62,044 | 53,780 | 46,724 | 61,047 | 53,166 | 45,958 | 60,771 | 52,311 | 44,016 | 60,994 | 51,703 | 41,864 | 62,012 |
| **F** | **80-84** | 46,119 | 36,978 | 55,553 | 46,728 | 39,011 | 54,355 | 46,928 | 40,370 | 53,318 | 46,816 | 40,470 | 52,998 | 46,870 | 39,683 | 53,956 | 46,896 | 38,413 | 55,341 |
| **F** | **85-89** | 29,824 | 24,462 | 35,710 | 30,566 | 26,237 | 35,542 | 31,611 | 27,854 | 35,963 | 32,818 | 28,920 | 37,235 | 34,040 | 29,342 | 39,056 | 34,547 | 29,014 | 40,741 |
| **F** | **90-94** | 12,677 | 10,498 | 15,018 | 13,839 | 11,807 | 16,074 | 14,544 | 12,681 | 16,570 | 15,010 | 13,137 | 17,043 | 15,508 | 13,367 | 17,917 | 15,777 | 13,178 | 18,611 |
| **F** | **95+** | 2,566 | 2,074 | 3,105 | 2,627 | 2,234 | 3,067 | 3,004 | 2,640 | 3,452 | 3,497 | 3,073 | 3,960 | 3,935 | 3,400 | 4,530 | 4,249 | 3,543 | 5,004 |
| **F** | **Total** | 549,362 | 511,107 | 587,842 | 572,010 | 540,979 | 605,278 | 594,621 | 565,549 | 624,761 | 616,704 | 588,534 | 647,924 | 638,614 | 602,717 | 676,131 | 653,496 | 609,968 | 700,830 |
| **F** | **Total per 100,000** | 9,699 | 9,024 | 10,378 | 10,053 | 9,507 | 10,637 | 10,401 | 9,892 | 10,928 | 10,740 | 10,249 | 11,283 | 11,396 | 10,636 | 12,221 | 11,396 | 10,636 | 12,221 |
| **M** | **<1** | - | - | - | - | - | - | - | - | - | - | - | - | - | - | - | - | - | - |
| **M** | **1-4** | - | - | - | - | - | - | - | - | - | - | - | - | - | - | - | - | - | - |
| **M** | **5-9** | - | - | - | - | - | - | - | - | - | - | - | - | - | - | - | - | - | - |
| **M** | **10-14** | - | - | - | - | - | - | - | - | - | - | - | - | - | - | - | - | - | - |
| **M** | **15-19** | 4,248 | 1,677 | 8,115 | 5,331 | 2,773 | 8,465 | 6,444 | 3,512 | 10,329 | 7,574 | 3,911 | 12,923 | 8,683 | 4,105 | 15,449 | 9,590 | 4,051 | 17,527 |
| **M** | **20-24** | 4,773 | 1,847 | 8,899 | 5,921 | 3,219 | 9,534 | 7,010 | 3,867 | 11,212 | 8,042 | 4,268 | 13,223 | 9,048 | 4,307 | 15,580 | 9,866 | 4,313 | 17,725 |
| **M** | **25-29** | 20,450 | 13,156 | 29,834 | 21,394 | 14,975 | 29,497 | 22,481 | 16,343 | 30,295 | 23,616 | 16,782 | 32,442 | 24,675 | 16,270 | 35,598 | 25,220 | 15,269 | 38,148 |
| **M** | **30-34** | 21,445 | 13,885 | 30,620 | 22,068 | 15,335 | 29,612 | 22,640 | 16,382 | 29,731 | 23,275 | 16,429 | 31,708 | 24,029 | 15,576 | 35,001 | 24,576 | 14,580 | 37,912 |
| **M** | **35-39** | 31,840 | 22,501 | 41,892 | 32,502 | 24,856 | 40,300 | 33,407 | 26,621 | 40,294 | 34,316 | 27,525 | 41,563 | 35,077 | 27,198 | 43,629 | 35,304 | 26,081 | 45,923 |
| **M** | **40-44** | 34,250 | 24,629 | 46,291 | 34,482 | 26,800 | 44,447 | 34,557 | 27,635 | 42,620 | 34,592 | 27,742 | 42,385 | 34,749 | 26,983 | 43,379 | 34,695 | 25,105 | 44,942 |
| **M** | **45-49** | 44,917 | 34,379 | 57,150 | 46,000 | 37,237 | 55,324 | 47,117 | 38,475 | 56,017 | 48,359 | 38,748 | 58,168 | 49,597 | 39,076 | 61,150 | 50,268 | 37,111 | 64,538 |
| **M** | **50-54** | 43,521 | 32,278 | 55,359 | 45,799 | 36,285 | 55,969 | 47,982 | 39,031 | 57,144 | 49,901 | 40,937 | 60,893 | 51,510 | 40,402 | 64,851 | 52,292 | 39,289 | 68,514 |
| **M** | **55-59** | 43,056 | 32,614 | 54,771 | 46,449 | 37,572 | 56,450 | 49,952 | 41,781 | 59,246 | 53,460 | 44,829 | 63,640 | 56,958 | 46,973 | 68,973 | 59,897 | 48,283 | 74,540 |
| **M** | **60-64** | 38,131 | 28,411 | 49,056 | 40,720 | 32,381 | 49,810 | 43,569 | 35,789 | 51,351 | 46,697 | 38,558 | 54,843 | 49,975 | 40,541 | 60,131 | 52,854 | 41,676 | 65,151 |
| **M** | **65-69** | 40,218 | 29,633 | 51,860 | 39,934 | 30,869 | 49,370 | 39,270 | 31,924 | 47,318 | 37,837 | 30,859 | 44,934 | 35,974 | 29,064 | 44,152 | 34,076 | 25,582 | 44,030 |
| **M** | **70-74** | 28,556 | 21,250 | 37,048 | 27,697 | 21,576 | 34,684 | 27,216 | 21,899 | 33,171 | 27,637 | 22,611 | 33,653 | 28,379 | 22,425 | 35,550 | 28,158 | 21,090 | 36,964 |
| **M** | **75-79** | 27,856 | 20,856 | 34,841 | 29,873 | 23,980 | 36,025 | 31,642 | 26,364 | 37,606 | 32,787 | 27,059 | 39,456 | 33,703 | 27,145 | 41,285 | 34,915 | 26,685 | 44,209 |
| **M** | **80-84** | 20,212 | 14,966 | 25,961 | 21,864 | 17,522 | 26,762 | 23,336 | 19,173 | 28,068 | 24,640 | 19,902 | 29,465 | 26,132 | 20,341 | 32,020 | 27,653 | 20,654 | 35,052 |
| **M** | **85-89** | 10,113 | 7,697 | 12,980 | 11,042 | 8,953 | 13,655 | 12,187 | 10,009 | 14,720 | 13,517 | 10,944 | 16,246 | 14,875 | 11,506 | 18,455 | 16,030 | 11,969 | 20,651 |
| **M** | **90-94** | 3,182 | 2,448 | 4,027 | 3,670 | 2,975 | 4,435 | 4,122 | 3,389 | 4,891 | 4,528 | 3,672 | 5,454 | 4,933 | 3,870 | 6,064 | 5,348 | 3,992 | 6,781 |
| **M** | **95+** | 386 | 290 | 496 | 437 | 349 | 533 | 555 | 454 | 662 | 707 | 573 | 851 | 856 | 669 | 1,051 | 975 | 710 | 1,241 |
| **M** | **Total** | 417,156 | 382,845 | 452,158 | 435,182 | 406,597 | 464,417 | 453,488 | 425,952 | 479,702 | 471,485 | 443,122 | 499,680 | 489,156 | 456,860 | 523,487 | 501,717 | 462,957 | 544,044 |
| **M** | **Total per 100,000** | 7,639 | 7,011 | 8,280 | 7,927 | 7,407 | 8,460 | 8,213 | 7,715 | 8,688 | 8,491 | 7,980 | 8,999 | 8,762 | 8,183 | 9,376 | 9,023 | 8,326 | 9,784 |

Appendix Table 2: YLDs of LBP and confidence intervals by sex, age and year

|  |  | **2013** | | | **2014** | | | **2015** | | | **2016** | | | **2017** | | | **2018** | | |
| --- | --- | --- | --- | --- | --- | --- | --- | --- | --- | --- | --- | --- | --- | --- | --- | --- | --- | --- | --- |
| **Sex** | **Age** | **YLD** | **2.5CI** | **97.5CI** | **YLD** | **2.5CI** | **97.5CI** | **YLD** | **2.5CI** | **97.5CI** | **YLD** | **2.5CI** | **97.5CI** | **YLD** | **2.5CI** | **97.5CI** | **YLD** | **2.5CI** | **97.5CI** |
| **F** | **<1** | - | - | - | - | - | - | - | - | - | - | - | - | - | - | - | - | - | - |
| **F** | **1-4** | - | - | - | - | - | - | - | - | - | - | - | - | - | - | - | - | - | - |
| **F** | **5-9** | - | - | - | - | - | - | - | - | - | - | - | - | - | - | - | - | - | - |
| **F** | **10-14** | - | - | - | - | - | - | - | - | - | - | - | - | - | - | - | - | - | - |
| **F** | **15-19** | 775 | 141 | 1,904 | 894 | 291 | 1,793 | 1,018 | 398 | 1,856 | 1,142 | 485 | 2,090 | 1,260 | 522 | 2,346 | 1,382 | 528 | 2,821 |
| **F** | **20-24** | 1,657 | 619 | 3,207 | 1,698 | 825 | 2,997 | 1,729 | 934 | 2,792 | 1,754 | 979 | 2,769 | 1,780 | 898 | 2,882 | 1,828 | 807 | 3,185 |
| **F** | **25-29** | 1,648 | 694 | 3,079 | 1,687 | 830 | 2,869 | 1,734 | 911 | 2,711 | 1,781 | 949 | 2,751 | 1,823 | 946 | 2,980 | 1,846 | 838 | 3,228 |
| **F** | **30-34** | 3,344 | 1,631 | 5,732 | 3,083 | 1,696 | 5,047 | 2,813 | 1,711 | 4,350 | 2,543 | 1,589 | 3,777 | 2,285 | 1,390 | 3,420 | 2,025 | 1,080 | 3,340 |
| **F** | **35-39** | 3,371 | 1,774 | 5,561 | 3,422 | 2,062 | 5,289 | 3,506 | 2,301 | 5,121 | 3,598 | 2,401 | 5,046 | 3,668 | 2,289 | 5,217 | 3,704 | 2,103 | 5,458 |
| **F** | **40-44** | 4,571 | 2,873 | 6,437 | 4,835 | 3,216 | 6,580 | 5,068 | 3,419 | 6,894 | 5,285 | 3,375 | 7,587 | 5,526 | 3,249 | 8,320 | 5,829 | 3,159 | 9,305 |
| **F** | **45-49** | 4,509 | 3,033 | 6,154 | 5,109 | 3,756 | 6,621 | 5,706 | 4,287 | 7,338 | 6,329 | 4,717 | 8,251 | 6,965 | 4,989 | 9,293 | 7,617 | 5,329 | 10,268 |
| **F** | **50-54** | 5,277 | 3,721 | 7,225 | 5,754 | 4,227 | 7,446 | 6,219 | 4,685 | 7,792 | 6,646 | 5,023 | 8,445 | 7,032 | 5,177 | 9,078 | 7,462 | 5,304 | 9,847 |
| **F** | **55-59** | 6,016 | 4,002 | 8,179 | 6,500 | 4,700 | 8,484 | 6,981 | 5,315 | 8,866 | 7,457 | 5,795 | 9,377 | 7,936 | 6,062 | 10,005 | 8,330 | 6,178 | 10,901 |
| **F** | **60-64** | 5,371 | 3,612 | 7,517 | 5,710 | 4,183 | 7,627 | 6,092 | 4,608 | 7,959 | 6,503 | 4,943 | 8,290 | 6,925 | 5,225 | 8,932 | 7,232 | 5,271 | 9,624 |
| **F** | **65-69** | 5,705 | 4,020 | 7,913 | 6,041 | 4,552 | 7,998 | 6,353 | 4,821 | 8,066 | 6,580 | 4,935 | 8,336 | 6,738 | 4,829 | 8,755 | 6,893 | 4,669 | 9,283 |
| **F** | **70-74** | 5,803 | 3,622 | 8,252 | 5,528 | 3,762 | 7,607 | 5,345 | 3,888 | 7,051 | 5,348 | 4,019 | 6,821 | 5,419 | 4,080 | 6,929 | 5,101 | 3,611 | 6,757 |
| **F** | **75-79** | 5,633 | 3,875 | 7,710 | 5,560 | 4,087 | 7,296 | 5,438 | 4,086 | 6,984 | 5,219 | 3,904 | 6,624 | 4,985 | 3,602 | 6,419 | 4,907 | 3,377 | 6,567 |
| **F** | **80-84** | 5,424 | 3,598 | 7,478 | 5,829 | 4,227 | 7,636 | 6,178 | 4,624 | 7,890 | 6,476 | 4,837 | 8,197 | 6,788 | 4,933 | 8,792 | 7,179 | 5,002 | 9,478 |
| **F** | **85-89** | 3,645 | 2,386 | 5,182 | 3,686 | 2,587 | 5,004 | 3,760 | 2,722 | 4,944 | 3,850 | 2,786 | 4,928 | 3,937 | 2,754 | 5,115 | 3,932 | 2,615 | 5,273 |
| **F** | **90-94** | 1,613 | 798 | 2,533 | 1,761 | 1,025 | 2,591 | 1,849 | 1,155 | 2,606 | 1,907 | 1,224 | 2,667 | 1,968 | 1,212 | 2,815 | 1,988 | 1,111 | 3,014 |
| **F** | **95+** | 334 | 92 | 647 | 287 | 84 | 544 | 267 | 88 | 486 | 240 | 77 | 468 | 192 | 54 | 438 | 117 | 2 | 394 |
| **F** | **Total** | 64,695 | 52,869 | 76,474 | 67,386 | 55,957 | 78,586 | 70,056 | 58,508 | 81,944 | 72,658 | 60,953 | 85,019 | 75,227 | 62,395 | 87,835 | 77,370 | 63,931 | 91,192 |
| **F** | **Total per 100,000** | 1,142 | 933 | 1,350 | 1,184 | 983 | 1,381 | 1,225 | 1,023 | 1433 | 1,265 | 1,061 | 1,481 | 1,305 | 1,082 | 1,523 | 1,343 | 1,109 | 1,583 |
| **F** | **Age-std per 100,000** | 1,117 |  |  | 1,154 |  |  | 1,192 |  |  | 1,229 |  |  | 1,266 |  |  | 1,303 |  |  |
| **M** | **<1** | - | - | - | - | - | - | - | - | - | - | - | - | - | - | - | - | - | - |
| **M** | **1-4** | - | - | - | - | - | - | - | - | - | - | - | - | - | - | - | - | - | - |
| **M** | **5-9** | - | - | - | - | - | - | - | - | - | - | - | - | - | - | - | - | - | - |
| **M** | **10-14** | - | - | - | - | - | - | - | - | - | - | - | - | - | - | - | - | - | - |
| **M** | **15-19** | 589 | 111 | 1,410 | 575 | 170 | 1,260 | 564 | 188 | 1,151 | 554 | 174 | 1,191 | 542 | 147 | 1,260 | 531 | 71 | 1,459 |
| **M** | **20-24** | 483 | 168 | 944 | 752 | 381 | 1,287 | 1,015 | 480 | 1,750 | 1,270 | 566 | 2,272 | 1,523 | 612 | 2,839 | 1,786 | 662 | 3,471 |
| **M** | **25-29** | 2,343 | 1,267 | 3,722 | 2,392 | 1,428 | 3,561 | 2,457 | 1,463 | 3,597 | 2,526 | 1,420 | 3,868 | 2,586 | 1,296 | 4,224 | 2,613 | 1,070 | 4,661 |
| **M** | **30-34** | 2,733 | 1,510 | 4,409 | 2,860 | 1,795 | 4,280 | 2,978 | 1,946 | 4,272 | 3,102 | 1,991 | 4,494 | 3,238 | 1,884 | 4,959 | 3,374 | 1,721 | 5,505 |
| **M** | **35-39** | 3,736 | 2,350 | 5,328 | 3,884 | 2,697 | 5,355 | 4,070 | 2,883 | 5,492 | 4,264 | 3,008 | 5,858 | 4,442 | 2,970 | 6,335 | 4,589 | 2,839 | 6,751 |
| **M** | **40-44** | 4,183 | 2,510 | 6,243 | 4,151 | 2,792 | 5,901 | 4,096 | 2,848 | 5,539 | 4,037 | 2,858 | 5,414 | 4,000 | 2,725 | 5,452 | 4,017 | 2,502 | 5,720 |
| **M** | **45-49** | 6,460 | 4,414 | 8,822 | 6,211 | 4,532 | 8,156 | 5,986 | 4,474 | 7,666 | 5,791 | 4,322 | 7,469 | 5,606 | 4,118 | 7,338 | 5,455 | 3,735 | 7,433 |
| **M** | **50-54** | 4,289 | 2,746 | 6,291 | 4,856 | 3,410 | 6,521 | 5,420 | 3,827 | 7,287 | 5,962 | 4,172 | 8,158 | 6,469 | 4,239 | 9,181 | 6,992 | 4,314 | 10,169 |
| **M** | **55-59** | 4,877 | 3,252 | 6,773 | 5,737 | 4,219 | 7,501 | 6,621 | 5,071 | 8,409 | 7,519 | 5,734 | 9,524 | 8,429 | 6,308 | 10,780 | 9,237 | 6,737 | 11,997 |
| **M** | **60-64** | 5,010 | 3,148 | 7,143 | 4,876 | 3,274 | 6,645 | 4,761 | 3,373 | 6,241 | 4,661 | 3,426 | 6,056 | 4,557 | 3,281 | 6,053 | 4,372 | 2,997 | 6,083 |
| **M** | **65-69** | 4,022 | 2,590 | 5,671 | 4,124 | 2,840 | 5,673 | 4,204 | 3,010 | 5,633 | 4,213 | 3,056 | 5,704 | 4,174 | 2,838 | 5,737 | 4,129 | 2,640 | 5,971 |
| **M** | **70-74** | 4,291 | 2,832 | 6,047 | 4,016 | 2,771 | 5,504 | 3,790 | 2,719 | 5,006 | 3,676 | 2,722 | 4,764 | 3,589 | 2,577 | 4,743 | 3,211 | 2,081 | 4,492 |
| **M** | **75-79** | 2,887 | 1,784 | 4,203 | 3,080 | 2,106 | 4,265 | 3,247 | 2,247 | 4,336 | 3,350 | 2,300 | 4,514 | 3,430 | 2,240 | 4,710 | 3,591 | 2,159 | 5,136 |
| **M** | **80-84** | 2,879 | 1,818 | 4,152 | 3,067 | 2,123 | 4,225 | 3,226 | 2,312 | 4,321 | 3,359 | 2,374 | 4,492 | 3,516 | 2,361 | 4,815 | 3,653 | 2,256 | 5,211 |
| **M** | **85-89** | 1,115 | 488 | 1,982 | 1,263 | 680 | 2,057 | 1,437 | 824 | 2,233 | 1,635 | 932 | 2,489 | 1,838 | 973 | 2,892 | 1,968 | 934 | 3,252 |
| **M** | **90-94** | 306 | 38 | 764 | 343 | 100 | 737 | 376 | 149 | 707 | 402 | 178 | 706 | 427 | 178 | 747 | 437 | 155 | 841 |
| **M** | **95+** | - | - | - | - | - | - | - | - | - | - | - | - | - | - | - | - | - | - |
| **M** | **Total** | 50,202 | 41,735 | 59,523 | 52,186 | 43,716 | 61,196 | 54,246 | 45,673 | 63,025 | 56,319 | 46,930 | 65,689 | 58,365 | 48,392 | 68,735 | 59,953 | 49,432 | 71,055 |
| **M** | **Total per 100,000** | 919 | 764 | 1,090 | 951 | 796 | 1,115 | 982 | 827 | 1,141 | 1,014 | 845 | 1,183 | 1,045 | 867 | 1,231 | 1,076 | 888 | 1,275 |
| **M** | **Age-std per 100,000** | 961 |  |  | 990 |  |  | 1,019 |  |  | 1,048 |  |  | 1,077 |  |  | 1,106 |  |  |

Appendix Table 3: NKP cases and confidence intervals by sex, age and year

|  |  | **2013** | | | **2014** | | | **2015** | | | **2016** | | | **2017** | | | **2018** | | |
| --- | --- | --- | --- | --- | --- | --- | --- | --- | --- | --- | --- | --- | --- | --- | --- | --- | --- | --- | --- |
| **Sex** | **Age** | **N** | **2.5CI** | **97.5CI** | **N** | **2.5CI** | **97.5CI** | **N** | **2.5CI** | **97.5CI** | **N** | **2.5CI** | **97.5CI** | **N** | **2.5CI** | **97.5CI** | **N** | **2.5CI** | **97.5CI** |
| **F** | **<1** | - | - | - | - | - | - | - | - | - | - | - | - | - | - | - | - | - | - |
| **F** | **1-4** | - | - | - | - | - | - | - | - | - | - | - | - | - | - | - | - | - | - |
| **F** | **5-9** | - | - | - | - | - | - | - | - | - | - | - | - | - | - | - | - | - | - |
| **F** | **10-14** | - | - | - | - | - | - | - | - | - | - | - | - | - | - | - | - | - | - |
| **F** | **15-19** | 5,998 | 2,066 | 11,729 | 6,325 | 3,104 | 11,104 | 6,687 | 3,623 | 10,992 | 7,039 | 3,657 | 11,779 | 7,367 | 3,471 | 13,005 | 7,676 | 3,166 | 14,610 |
| **F** | **20-24** | 6,793 | 2,764 | 13,916 | 7,179 | 3,584 | 12,779 | 7,510 | 4,023 | 12,569 | 7,806 | 4,191 | 13,110 | 8,099 | 3,950 | 14,677 | 8,367 | 3,423 | 16,386 |
| **F** | **25-29** | 8,423 | 4,698 | 13,486 | 9,410 | 6,236 | 13,687 | 10,469 | 7,175 | 14,450 | 11,554 | 7,800 | 16,076 | 12,601 | 7,937 | 18,451 | 13,328 | 7,695 | 20,273 |
| **F** | **30-34** | 8,775 | 4,702 | 14,103 | 9,655 | 6,260 | 14,112 | 10,511 | 7,298 | 14,419 | 11,372 | 7,630 | 15,708 | 12,296 | 7,823 | 17,810 | 13,106 | 7,771 | 19,710 |
| **F** | **35-39** | 21,908 | 13,516 | 32,019 | 23,866 | 16,568 | 32,882 | 26,028 | 18,118 | 34,515 | 28,255 | 18,909 | 38,903 | 30,355 | 19,365 | 42,846 | 31,896 | 18,454 | 47,709 |
| **F** | **40-44** | 23,429 | 14,193 | 35,353 | 25,181 | 16,964 | 34,673 | 26,778 | 18,370 | 35,626 | 28,277 | 19,194 | 38,701 | 29,842 | 18,976 | 42,878 | 31,151 | 18,230 | 47,878 |
| **F** | **45-49** | 42,303 | 32,045 | 53,194 | 45,110 | 36,795 | 54,226 | 47,916 | 40,491 | 56,473 | 50,925 | 43,006 | 60,510 | 54,011 | 44,859 | 65,261 | 56,313 | 45,034 | 69,650 |
| **F** | **50-54** | 41,997 | 32,382 | 53,771 | 45,862 | 37,245 | 55,869 | 49,636 | 41,524 | 58,869 | 53,107 | 44,088 | 63,177 | 56,244 | 45,300 | 68,244 | 58,534 | 45,391 | 73,162 |
| **F** | **55-59** | 37,106 | 27,380 | 47,100 | 39,596 | 31,346 | 48,060 | 42,074 | 33,986 | 49,778 | 44,518 | 36,755 | 52,699 | 46,974 | 37,874 | 56,622 | 48,929 | 37,584 | 60,414 |
| **F** | **60-64** | 33,464 | 25,407 | 42,385 | 35,381 | 28,312 | 42,790 | 37,559 | 31,604 | 44,834 | 39,926 | 33,758 | 47,567 | 42,373 | 35,014 | 51,449 | 44,397 | 35,754 | 55,687 |
| **F** | **65-69** | 34,225 | 24,756 | 44,487 | 36,619 | 28,325 | 45,427 | 38,917 | 31,390 | 46,610 | 40,709 | 32,706 | 48,773 | 42,057 | 33,128 | 51,434 | 43,047 | 33,018 | 54,292 |
| **F** | **70-74** | 26,495 | 18,687 | 34,987 | 27,426 | 21,242 | 34,403 | 28,836 | 23,582 | 34,813 | 31,462 | 25,795 | 37,516 | 34,877 | 27,640 | 42,509 | 37,438 | 28,189 | 47,146 |
| **F** | **75-79** | 31,460 | 23,416 | 40,710 | 32,368 | 25,841 | 40,007 | 32,997 | 27,543 | 39,163 | 32,992 | 27,536 | 38,785 | 32,819 | 26,747 | 39,888 | 32,816 | 25,649 | 41,136 |
| **F** | **80-84** | 27,675 | 21,127 | 35,363 | 28,348 | 23,103 | 34,739 | 28,790 | 24,033 | 34,314 | 29,048 | 24,252 | 34,605 | 29,413 | 23,919 | 35,625 | 29,791 | 22,796 | 37,306 |
| **F** | **85-89** | 17,933 | 13,729 | 22,644 | 18,558 | 15,164 | 22,779 | 19,385 | 16,057 | 22,900 | 20,333 | 16,793 | 24,211 | 21,314 | 17,331 | 25,731 | 21,891 | 17,085 | 27,324 |
| **F** | **90-94** | 7,634 | 5,840 | 9,622 | 8,406 | 6,827 | 10,126 | 8,915 | 7,350 | 10,500 | 9,286 | 7,617 | 10,969 | 9,687 | 7,809 | 11,600 | 9,963 | 7,692 | 12,461 |
| **F** | **95+** | 793 | 1,181 | 1,907 | 1,595 | 1,293 | 1,896 | 1,841 | 1,539 | 2,132 | 2,161 | 1,803 | 2,514 | 2,452 | 2,000 | 2,922 | 2,674 | 2,086 | 3,310 |
| **F** | **Total** | 376,410 | 345,754 | 410,618 | 400,885 | 371,471 | 426,114 | 424,849 | 400,467 | 448,669 | 448,769 | 422,077 | 475,663 | 472,781 | 439,997 | 504,885 | 491,317 | 452,097 | 531,225 |
| **F** | **Total per 100,000** | 6,645 | 6,104 | 7,249 | 7,045 | 6,528 | 7,489 | 7,431 | 7,005 | 7,848 | 7,815 | 7,350 | 8,284 | 8,199 | 7,631 | 8,756 | 8,567 | 7,884 | 9,263 |
| **M** | **<1** | - | - | - | - | - | - | - | - | - | - | - | - | - | - | - | - | - | - |
| **M** | **1-4** | - | - | - | - | - | - | - | - | - | - | - | - | - | - | - | - | - | - |
| **M** | **5-9** | - | - | - | - | - | - | - | - | - | - | - | - | - | - | - | - | - | - |
| **M** | **10-14** | - | - | - | - | - | - | - | - | - | - | - | - | - | - | - | - | - | - |
| **M** | **15-19** | 3,438 | 894 | 7,919 | 3,734 | 1,480 | 7,449 | 4,058 | 1,705 | 7,779 | 4,403 | 1,711 | 8,850 | 4,745 | 1,570 | 10,309 | 4,950 | 1,215 | 11,890 |
| **M** | **20-24** | 3,755 | 953 | 8,492 | 4,087 | 1,649 | 8,031 | 4,372 | 1,958 | 8,272 | 4,629 | 1,939 | 9,081 | 4,877 | 1,805 | 10,480 | 4,999 | 1,298 | 11,725 |
| **M** | **25-29** | 5,369 | 2,194 | 10,862 | 5,956 | 3,028 | 10,641 | 6,576 | 3,518 | 10,913 | 7,218 | 3,629 | 11,945 | 7,854 | 3,522 | 13,922 | 8,369 | 3,164 | 15,750 |
| **M** | **30-34** | 5,558 | 2,240 | 10,749 | 6,091 | 3,135 | 10,636 | 6,606 | 3,544 | 10,824 | 7,125 | 3,599 | 12,333 | 7,670 | 3,530 | 14,000 | 8,167 | 3,198 | 15,696 |
| **M** | **35-39** | 11,710 | 7,045 | 17,784 | 12,976 | 8,967 | 18,160 | 14,349 | 10,416 | 18,975 | 15,751 | 11,094 | 21,070 | 17,113 | 11,395 | 23,870 | 18,229 | 11,328 | 26,379 |
| **M** | **40-44** | 12,571 | 7,427 | 19,424 | 13,761 | 9,508 | 19,329 | 14,852 | 10,776 | 19,809 | 15,883 | 11,057 | 21,378 | 16,943 | 11,036 | 23,797 | 17,896 | 10,883 | 26,221 |
| **M** | **45-49** | 24,172 | 16,669 | 32,880 | 24,858 | 18,541 | 32,138 | 25,509 | 19,791 | 32,234 | 26,159 | 19,517 | 34,178 | 26,739 | 18,963 | 36,728 | 27,096 | 17,725 | 39,568 |
| **M** | **50-54** | 23,119 | 16,019 | 31,485 | 24,474 | 18,368 | 31,865 | 25,780 | 19,815 | 32,413 | 26,934 | 20,274 | 34,586 | 27,892 | 19,691 | 38,196 | 28,497 | 18,557 | 40,768 |
| **M** | **55-59** | 24,497 | 16,518 | 34,788 | 26,001 | 18,900 | 34,746 | 27,528 | 21,090 | 35,385 | 29,045 | 21,943 | 36,636 | 30,554 | 22,693 | 39,670 | 31,782 | 22,434 | 41,961 |
| **M** | **60-64** | 21,686 | 14,076 | 30,566 | 22,787 | 16,435 | 30,151 | 24,021 | 17,897 | 30,439 | 25,386 | 19,018 | 32,020 | 26,806 | 19,566 | 34,535 | 28,013 | 19,774 | 37,439 |
| **M** | **65-69** | 24,963 | 15,978 | 35,800 | 23,383 | 16,187 | 32,374 | 21,525 | 15,345 | 28,462 | 19,200 | 13,760 | 24,845 | 16,621 | 11,595 | 22,056 | 13,967 | 8,383 | 20,383 |
| **M** | **70-74** | 17,812 | 11,142 | 25,397 | 16,268 | 10,884 | 22,495 | 14,908 | 10,345 | 19,901 | 13,950 | 10,065 | 18,323 | 13,019 | 9,327 | 17,467 | 11,477 | 7,157 | 16,651 |
| **M** | **75-79** | 17,173 | 10,808 | 24,312 | 16,896 | 11,700 | 22,503 | 16,485 | 12,123 | 21,539 | 15,778 | 11,918 | 20,470 | 15,007 | 10,859 | 20,413 | 14,317 | 9,429 | 20,542 |
| **M** | **80-84** | 12,496 | 8,252 | 18,198 | 12,405 | 8,930 | 17,094 | 12,166 | 9,240 | 15,940 | 11,822 | 8,802 | 15,602 | 11,551 | 8,278 | 15,693 | 11,215 | 7,212 | 16,330 |
| **M** | **85-89** | 6,281 | 4,112 | 8,901 | 6,302 | 4,434 | 8,480 | 6,399 | 4,728 | 8,266 | 6,535 | 4,882 | 8,418 | 6,635 | 4,735 | 8,887 | 6,559 | 4,380 | 9,529 |
| **M** | **90-94** | 1,989 | 1,296 | 2,730 | 2,105 | 1,489 | 2,786 | 2,173 | 1,610 | 2,771 | 2,195 | 1,611 | 2,816 | 2,201 | 1,558 | 2,970 | 2,188 | 1,393 | 3,177 |
| **M** | **95+** | 243 | 161 | 336 | 253 | 183 | 335 | 296 | 223 | 378 | 346 | 259 | 443 | 385 | 279 | 514 | 399 | 261 | 571 |
| **M** | **Total** | 216,832 | 192,862 | 241,575 | 222,335 | 202,901 | 242,626 | 227,603 | 208,800 | 246,398 | 232,362 | 213,047 | 252,471 | 236,612 | 214,591 | 261,142 | 238,121 | 212,277 | 268,174 |
| **M** | **Total per 100,000** | 3,971 | 3,532 | 4,424 | 4,050 | 3,696 | 4,420 | 4,122 | 3,782 | 4,463 | 4,185 | 3,837 | 4,547 | 4,238 | 3,844 | 4,677 | 4,282 | 3,818 | 4,823 |

Appendix Table 4: YLDs of NKP by sex, age and year

|  |  | **2013** | | | **2014** | | | **2015** | | | **2016** | | | **2017** | | | **2018** | | |
| --- | --- | --- | --- | --- | --- | --- | --- | --- | --- | --- | --- | --- | --- | --- | --- | --- | --- | --- | --- |
| **Sex** | **Age** | **YLD** | **2.5CI** | **97.5CI** | **YLD** | **2.5CI** | **97.5CI** | **YLD** | **2.5CI** | **97.5CI** | **YLD** | **2.5CI** | **97.5CI** | **YLD** | **2.5CI** | **97.5CI** | **YLD** | **2.5CI** | **97.5CI** |
| **F** | **<1** | - | - | - | - | - | - | - | - | - | - | - | - | - | - | - | - | - | - |
| **F** | **1-4** | - | - | - | - | - | - | - | - | - | - | - | - | - | - | - | - | - | - |
| **F** | **5-9** | - | - | - | - | - | - | - | - | - | - | - | - | - | - | - | - | - | - |
| **F** | **10-14** | - | - | - | - | - | - | - | - | - | - | - | - | - | - | - | - | - | - |
| **F** | **15-19** | 47 | 6 | 131 | 175 | 49 | 402 | 305 | 71 | 765 | 435 | 89 | 1,125 | 562 | 103 | 1,483 | 691 | 123 | 1,852 |
| **F** | **20-24** | 1,270 | 446 | 2,435 | 1,210 | 535 | 2,184 | 1,143 | 557 | 1,970 | 1,075 | 532 | 1,834 | 1,010 | 465 | 1,811 | 958 | 333 | 1,915 |
| **F** | **25-29** | 885 | 270 | 1,945 | 955 | 390 | 1,834 | 1,031 | 496 | 1,810 | 1,108 | 517 | 1,899 | 1,182 | 520 | 2,069 | 1,246 | 469 | 2,294 |
| **F** | **30-34** | 872 | 390 | 1,560 | 995 | 534 | 1,589 | 1,113 | 617 | 1,722 | 1,230 | 670 | 1,960 | 1,352 | 676 | 2,266 | 1,474 | 701 | 2,598 |
| **F** | **35-39** | 2,142 | 852 | 4,164 | 2,156 | 1,046 | 3,800 | 2,190 | 1,224 | 3,539 | 2,228 | 1,297 | 3,390 | 2,253 | 1,334 | 3,406 | 2,256 | 1,249 | 3,554 |
| **F** | **40-44** | 2,485 | 1,397 | 4,057 | 2,840 | 1,709 | 4,234 | 3,170 | 1,925 | 4,798 | 3,484 | 1,994 | 5,466 | 3,809 | 2,061 | 6,353 | 4,175 | 2,003 | 7,175 |
| **F** | **45-49** | 3,886 | 2,351 | 6,005 | 4,310 | 2,865 | 6,320 | 4,735 | 3,225 | 6,670 | 5,182 | 3,487 | 7,292 | 5,640 | 3,737 | 8,116 | 6,112 | 3,847 | 8,896 |
| **F** | **50-54** | 4,836 | 3,057 | 7,029 | 4,997 | 3,380 | 7,067 | 5,143 | 3,615 | 7,086 | 5,256 | 3,739 | 7,122 | 5,340 | 3,766 | 7,315 | 5,457 | 3,692 | 7,682 |
| **F** | **55-59** | 3,886 | 2,292 | 5,785 | 4,252 | 2,785 | 6,025 | 4,616 | 3,206 | 6,338 | 4,979 | 3,486 | 6,783 | 5,345 | 3,734 | 7,343 | 5,654 | 3,817 | 7,946 |
| **F** | **60-64** | 3,247 | 2,047 | 4,850 | 3,339 | 2,224 | 4,758 | 3,453 | 2,368 | 4,740 | 3,581 | 2,489 | 4,892 | 3,710 | 2,521 | 5,141 | 3,777 | 2,486 | 5,445 |
| **F** | **65-69** | 3,311 | 2,022 | 5,144 | 3,525 | 2,308 | 5,137 | 3,725 | 2,506 | 5,240 | 3,877 | 2,607 | 5,370 | 3,988 | 2,579 | 5,780 | 4,097 | 2,466 | 6,130 |
| **F** | **70-74** | 2,754 | 1,555 | 4,243 | 2,874 | 1,829 | 4,218 | 3,046 | 2,070 | 4,286 | 3,345 | 2,338 | 4,610 | 3,725 | 2,571 | 5,119 | 3,863 | 2,512 | 5,470 |
| **F** | **75-79** | 3,202 | 1,855 | 4,881 | 3,008 | 1,855 | 4,455 | 2,789 | 1,804 | 4,059 | 2,525 | 1,623 | 3,654 | 2,263 | 1,380 | 3,401 | 2,076 | 1,092 | 3,308 |
| **F** | **80-84** | 2,269 | 1,221 | 3,561 | 2,784 | 1,799 | 4,135 | 3,269 | 2,238 | 4,630 | 3,720 | 2,538 | 5,269 | 4,171 | 2,765 | 5,965 | 4,668 | 3,015 | 6,796 |
| **F** | **85-89** | 1,951 | 967 | 3,261 | 1,936 | 1,096 | 3,095 | 1,936 | 1,184 | 2,958 | 1,944 | 1,213 | 2,932 | 1,948 | 1,187 | 2,981 | 1,905 | 1,062 | 3,100 |
| **F** | **90-94** | 1,002 | 357 | 1,967 | 1,091 | 523 | 1,962 | 1,142 | 613 | 1,888 | 1,174 | 649 | 1,823 | 1,208 | 637 | 1,964 | 1,217 | 543 | 2,054 |
| **F** | **95+** | 244 | 36 | 562 | 212 | 41 | 465 | 201 | 44 | 424 | 186 | 41 | 392 | 157 | 30 | 401 | 106 | 2 | 391 |
| **F** | **Total** | 38,289 | 28,808 | 49,354 | 40,658 | 31,108 | 52,037 | 43,008 | 32,818 | 54,939 | 45,330 | 34,435 | 57,597 | 47,664 | 35,994 | 60,573 | 49,731 | 37,363 | 63,419 |
| **F** | **Total per 100,000** | 676 | 509 | 871 | 715 | 547 | 915 | 752 | 574 | 961 | 789 | 600 | 1,003 | 827 | 624 | 1,050 | 864 | 649 | 1,101 |
| **F** | **Age-std per 100,000** | 663 |  |  | 699 |  |  | 735 |  |  | 771 |  |  | 807 |  |  | 842 |  |  |
| **M** | **<1** | - | - | - | - | - | - | - | - | - | - | - | - | - | - | - | - | - | - |
| **M** | **1-4** | - | - | - | - | - | - | - | - | - | - | - | - | - | - | - | - | - | - |
| **M** | **5-9** | - | - | - | - | - | - | - | - | - | - | - | - | - | - | - | - | - | - |
| **M** | **10-14** | - | - | - | - | - | - | - | - | - | - | - | - | - | - | - | - | - | - |
| **M** | **15-19** | 477 | 60 | 1,348 | 438 | 85 | 1,124 | 401 | 83 | 974 | 364 | 70 | 875 | 327 | 49 | 956 | 289 | 8 | 1,056 |
| **M** | **20-24** | 247 | 47 | 630 | 363 | 116 | 777 | 476 | 134 | 1,041 | 586 | 144 | 1,377 | 694 | 135 | 1,743 | 807 | 120 | 2,113 |
| **M** | **25-29** | 496 | 129 | 1,239 | 460 | 152 | 1,065 | 425 | 144 | 920 | 390 | 127 | 838 | 352 | 95 | 874 | 309 | 30 | 945 |
| **M** | **30-34** | 659 | 155 | 1,556 | 793 | 307 | 1,609 | 924 | 403 | 1,723 | 1,056 | 463 | 2,021 | 1,191 | 483 | 2,336 | 1,327 | 444 | 2,689 |
| **M** | **35-39** | 1,740 | 863 | 3,049 | 1,659 | 927 | 2,760 | 1,592 | 942 | 2,597 | 1,525 | 917 | 2,447 | 1,450 | 811 | 2,408 | 1,363 | 648 | 2,501 |
| **M** | **40-44** | 614 | 267 | 1,149 | 953 | 561 | 1,477 | 1,277 | 755 | 2,000 | 1,587 | 915 | 2,558 | 1,895 | 1,054 | 3,107 | 2,224 | 1,152 | 3,747 |
| **M** | **45-49** | 2,366 | 1,327 | 3,671 | 2,220 | 1,326 | 3,274 | 2,085 | 1,325 | 2,982 | 1,961 | 1,249 | 2,875 | 1,841 | 1,096 | 2,819 | 1,733 | 935 | 2,831 |
| **M** | **50-54** | 2,481 | 1,422 | 3,892 | 2,769 | 1,783 | 4,044 | 3,056 | 2,016 | 4,435 | 3,329 | 2,113 | 4,997 | 3,583 | 2,145 | 5,660 | 3,846 | 2,112 | 6,362 |
| **M** | **55-59** | 2,416 | 1,411 | 3,857 | 2,698 | 1,759 | 4,041 | 2,987 | 2,010 | 4,266 | 3,278 | 2,216 | 4,691 | 3,572 | 2,313 | 5,222 | 3,821 | 2,369 | 5,813 |
| **M** | **60-64** | 2,220 | 1,063 | 3,866 | 2,188 | 1,173 | 3,566 | 2,166 | 1,260 | 3,336 | 2,151 | 1,281 | 3,319 | 2,136 | 1,265 | 3,444 | 2,083 | 1,101 | 3,625 |
| **M** | **65-69** | 2,117 | 1,078 | 3,504 | 1,959 | 1,040 | 3,157 | 1,775 | 1,015 | 2,813 | 1,552 | 910 | 2,400 | 1,309 | 745 | 2,072 | 1,062 | 458 | 1,898 |
| **M** | **70-74** | 2,190 | 1,113 | 3,709 | 2,011 | 1,129 | 3,313 | 1,855 | 1,099 | 2,951 | 1,751 | 1,071 | 2,700 | 1,651 | 978 | 2,596 | 1,414 | 718 | 2,440 |
| **M** | **75-79** | 1,538 | 773 | 2,539 | 1,511 | 848 | 2,382 | 1,471 | 863 | 2,233 | 1,404 | 818 | 2,171 | 1,331 | 702 | 2,187 | 1,293 | 585 | 2,302 |
| **M** | **80-84** | 1,409 | 644 | 2,548 | 1,514 | 820 | 2,508 | 1,604 | 955 | 2,477 | 1,681 | 1,005 | 2,639 | 1,771 | 1,010 | 2,789 | 1,850 | 959 | 3,073 |
| **M** | **85-89** | 743 | 241 | 1,490 | 632 | 223 | 1,256 | 520 | 191 | 1,021 | 399 | 164 | 756 | 264 | 114 | 481 | 110 | 27 | 273 |
| **M** | **90-94** | 114 | 2 | 393 | 129 | 19 | 374 | 142 | 31 | 348 | 152 | 38 | 349 | 162 | 41 | 371 | 167 | 36 | 414 |
| **M** | **95+** | - | - | - | - | - | - | - | - | - | - | - | - | - | - | - | - | - | - |
| **M** | **Total** | 21,827 | 16,272 | 28,813 | 22,296 | 16,635 | 29,062 | 22,754 | 16,927 | 29,125 | 23,165 | 17,190 | 29,760 | 23,527 | 17,294 | 30,338 | 23,697 | 17,022 | 31,111 |
| **M** | **Total per 100,000** | 400 | 298 | 528 | 406 | 303 | 529 | 412 | 307 | 528 | 417 | 310 | 536 | 421 | 310 | 543 | 425 | 306 | 558 |
| **M** | **Age-std per 100,000** | 426 |  |  | 428 |  |  | 429 |  |  | 430 |  |  | 431 |  |  | 433 |  |  |

Appendix Table 5: Number of OST cases by sex, age and year

|  |  | **2013** | | | | **2014** | | | **2015** | | | | **2016** | | | | | **2017** | | | **2018** | | | |  |
| --- | --- | --- | --- | --- | --- | --- | --- | --- | --- | --- | --- | --- | --- | --- | --- | --- | --- | --- | --- | --- | --- | --- | --- | --- | --- |
| **Sex** | **Age** | **N** | **2.5CI** | **97.5CI** | **N** | | **2.5CI** | **97.5CI** | | **N** | **2.5CI** | **97.5CI** | | **N** | **2.5CI** | **97.5CI** | **N** | | **2.5CI** | **97.5CI** | | **N** | **2.5CI** | **97.5CI** | |
| **F** | **<1** | - | - | - | - | | - | - | | - | - | - | | - | - | - | - | | - | - | | - | - | - | |
| **F** | **1-4** | - | - | - | - | | - | - | | - | - | - | | - | - | - | - | | - | - | | - | - | - | |
| **F** | **5-9** | - | - | - | - | | - | - | | - | - | - | | - | - | - | - | | - | - | | - | - | - | |
| **F** | **10-14** | - | - | - | - | | - | - | | - | - | - | | - | - | - | - | | - | - | | - | - | - | |
| **F** | **15-19** | 1,724 | 199 | 5,237 | 1,752 | | 867 | 5,382 | | 2,736 | 1,256 | 6,433 | | 3,709 | 1,498 | 8,414 | 4,656 | | 1,643 | 10,430 | | 5,582 | 1,744 | 12,508 | |
| **F** | **20-24** | 1,900 | 259 | 5,392 | 1,929 | | 1,138 | 5,773 | | 2,989 | 1,620 | 7,159 | | 4,023 | 1,873 | 8,970 | 5,040 | | 1,960 | 11,189 | | 6,025 | 2,025 | 13,473 | |
| **F** | **25-29** | 13,459 | 6,895 | 22,630 | 9,817 | | 7,502 | 20,730 | | 10,238 | 7,876 | 18,994 | | 10,672 | 7,680 | 17,772 | 11,079 | | 7,089 | 18,258 | | 11,300 | 5,594 | 19,226 | |
| **F** | **30-34** | 14,048 | 6,982 | 25,041 | 10,043 | | 7,649 | 22,017 | | 10,316 | 7,758 | 19,200 | | 10,581 | 7,520 | 17,914 | 10,894 | | 6,938 | 18,207 | | 11,153 | 5,382 | 18,753 | |
| **F** | **35-39** | 22,320 | 15,197 | 29,711 | 24,241 | | 17,949 | 30,643 | | 25,933 | 19,802 | 32,875 | | 27,727 | 20,829 | 35,824 | 29,419 | | 20,837 | 39,438 | | 30,557 | 20,208 | 42,749 | |
| **F** | **40-44** | 24,086 | 17,279 | 32,498 | 25,972 | | 19,357 | 32,151 | | 27,081 | 20,495 | 33,916 | | 28,083 | 20,442 | 36,239 | 29,155 | | 20,156 | 39,301 | | 30,001 | 19,794 | 42,134 | |
| **F** | **45-49** | 71,098 | 57,024 | 86,767 | 70,663 | | 61,356 | 85,441 | | 72,452 | 64,472 | 84,365 | | 74,722 | 66,250 | 85,947 | 77,226 | | 67,040 | 88,714 | | 78,557 | 66,202 | 91,714 | |
| **F** | **50-54** | 70,883 | 56,712 | 86,820 | 72,274 | | 61,993 | 87,461 | | 75,378 | 65,901 | 88,490 | | 78,013 | 68,599 | 90,457 | 80,209 | | 69,051 | 93,145 | | 81,129 | 66,739 | 96,340 | |
| **F** | **55-59** | 118,201 | 102,890 | 135,462 | 137,436 | | 109,937 | 136,768 | | 137,444 | 115,178 | 138,159 | | 137,081 | 118,162 | 141,602 | 136,526 | | 119,802 | 147,055 | | 134,251 | 117,981 | 151,332 | |
| **F** | **60-64** | 106,331 | 90,439 | 121,657 | 122,500 | | 96,022 | 121,276 | | 122,610 | 100,825 | 123,201 | | 123,000 | 104,708 | 126,705 | 123,266 | | 106,734 | 132,650 | | 121,961 | 106,381 | 138,190 | |
| **F** | **65-69** | 133,347 | 116,937 | 151,220 | 135,197 | | 123,688 | 152,969 | | 140,001 | 129,417 | 155,409 | | 142,780 | 131,544 | 155,886 | 143,981 | | 131,601 | 157,238 | | 144,123 | 127,633 | 158,483 | |
| **F** | **70-74** | 103,266 | 89,301 | 117,081 | 101,206 | | 91,875 | 114,663 | | 103,842 | 95,808 | 115,283 | | 110,635 | 102,443 | 121,966 | 119,736 | | 110,169 | 131,106 | | 125,601 | 112,841 | 138,650 | |
| **F** | **75-79** | 119,266 | 107,664 | 129,824 | 119,895 | | 108,302 | 126,784 | | 116,766 | 106,646 | 123,545 | | 111,565 | 102,017 | 119,081 | 106,083 | | 96,607 | 114,969 | | 101,404 | 91,301 | 112,528 | |
| **F** | **80-84** | 104,496 | 94,412 | 115,105 | 104,622 | | 94,920 | 111,931 | | 101,770 | 93,580 | 108,022 | | 98,218 | 90,751 | 104,912 | 95,056 | | 86,955 | 103,148 | | 91,915 | 82,744 | 102,008 | |
| **F** | **85-89** | 67,305 | 60,242 | 73,544 | 67,829 | | 61,516 | 72,443 | | 68,170 | 62,281 | 72,394 | | 68,690 | 62,929 | 73,322 | 69,023 | | 62,885 | 74,925 | | 67,810 | 60,578 | 75,393 | |
| **F** | **90-94** | 28,490 | 25,693 | 31,207 | 30,387 | | 27,972 | 32,784 | | 31,170 | 28,890 | 33,388 | | 31,346 | 29,206 | 33,474 | 31,496 | | 29,004 | 34,041 | | 31,136 | 28,189 | 34,455 | |
| **F** | **95+** | 5,749 | 5,230 | 6,278 | 5,710 | | 5,336 | 6,192 | | 6,403 | 6,015 | 6,866 | | 7,292 | 6,847 | 7,826 | 8,019 | | 7,409 | 8,668 | | 8,462 | 7,648 | 9,334 | |
| **F** | **Total** | 1,005,971 | 961,227 | 1,051,927 | 1,041,472 | | 985,375 | 1,062,057 | | 1,055,300 | 1,010,145 | 1,078,012 | | 1,068,137 | 1,027,810 | 1,094,063 | 1,080,864 | | 1,040,088 | 1,112,662 | | 1,080,969 | 1,039,022 | 1,124,081 | |
| **F** | **Total per 100,000** | 17,760 | 16,970 | 18,572 | 18,303 | | 17,317 | 18,665 | | 18,458 | 17,669 | 18,856 | | 18,601 | 17,899 | 19,053 | 18,745 | | 18,038 | 19,296 | | 18,850 | 18,118 | 19,601 | |
| **M** | **<1** | - | - | - | - | | - | - | | - | - | - | | - | - | - | - | | - | - | | - | - | - | |
| **M** | **1-4** | - | - | - | - | | - | - | | - | - | - | | - | - | - | - | | - | - | | - | - | - | |
| **M** | **5-9** | - | - | - | - | | - | - | | - | - | - | | - | - | - | - | | - | - | | - | - | - | |
| **M** | **10-14** | - | - | - | - | | - | - | | - | - | - | | - | - | - | - | | - | - | | - | - | - | |
| **M** | **15-19** | - | - | - | 676 | | 5 | 706 | | 755 | 9 | 1,421 | | 835 | 14 | 2,148 | 913 | | 19 | 2,874 | | 959 | 23 | 3,488 | |
| **M** | **20-24** | - | - | - | 725 | | 5 | 805 | | 791 | 10 | 1,585 | | 857 | 15 | 2,346 | 921 | | 19 | 3,088 | | 959 | 23 | 3,714 | |
| **M** | **25-29** | 9,266 | 3,885 | 17,272 | 10,558 | | 3,786 | 14,378 | | 8,939 | 3,706 | 11,756 | | 7,273 | 3,185 | 9,437 | 5,526 | | 2,489 | 7,968 | | 3,679 | 1,275 | 7,601 | |
| **M** | **30-34** | 9,655 | 3,641 | 18,528 | 10,862 | | 3,527 | 15,697 | | 9,005 | 3,477 | 12,685 | | 7,187 | 3,011 | 10,295 | 5,401 | | 2,429 | 8,283 | | 3,595 | 1,258 | 7,395 | |
| **M** | **35-39** | 17,497 | 10,706 | 25,918 | 20,593 | | 13,867 | 26,817 | | 22,649 | 16,266 | 28,680 | | 24,805 | 17,779 | 32,385 | 26,915 | | 18,644 | 35,875 | | 28,648 | 18,574 | 39,964 | |
| **M** | **40-44** | 19,210 | 12,077 | 28,305 | 22,310 | | 15,302 | 29,031 | | 23,808 | 17,316 | 30,388 | | 25,215 | 18,044 | 32,488 | 26,701 | | 18,473 | 35,722 | | 28,097 | 18,326 | 39,222 | |
| **M** | **45-49** | 46,238 | 35,114 | 58,638 | 55,780 | | 38,736 | 57,427 | | 55,256 | 40,898 | 58,657 | | 55,000 | 41,607 | 61,339 | 54,830 | | 41,298 | 64,893 | | 53,988 | 40,754 | 68,524 | |
| **M** | **50-54** | 45,181 | 34,282 | 57,372 | 56,085 | | 38,362 | 58,442 | | 56,719 | 41,399 | 60,790 | | 57,044 | 42,536 | 63,893 | 57,046 | | 42,645 | 68,107 | | 56,088 | 41,285 | 71,482 | |
| **M** | **55-59** | 76,614 | 62,930 | 92,328 | 87,920 | | 68,833 | 92,935 | | 90,085 | 73,883 | 95,125 | | 92,138 | 77,324 | 98,738 | 94,076 | | 79,443 | 104,708 | | 95,072 | 79,400 | 110,940 | |
| **M** | **60-64** | 67,852 | 55,358 | 81,126 | 77,276 | | 60,666 | 81,967 | | 78,815 | 64,710 | 84,566 | | 80,714 | 68,191 | 87,937 | 82,729 | | 70,402 | 92,846 | | 83,994 | 71,266 | 99,132 | |
| **M** | **65-69** | 71,863 | 58,977 | 86,822 | 83,167 | | 64,630 | 89,025 | | 86,069 | 70,233 | 91,493 | | 87,322 | 73,381 | 94,413 | 87,370 | | 74,143 | 97,356 | | 86,775 | 73,552 | 100,604 | |
| **M** | **70-74** | 51,589 | 41,811 | 61,658 | 56,640 | | 45,060 | 61,656 | | 58,222 | 48,608 | 62,613 | | 62,167 | 53,213 | 67,582 | 67,572 | | 57,671 | 75,359 | | 70,866 | 59,671 | 82,079 | |
| **M** | **75-79** | 52,906 | 43,468 | 63,764 | 66,392 | | 47,129 | 64,125 | | 65,108 | 49,839 | 64,456 | | 62,613 | 50,159 | 64,607 | 59,854 | | 49,093 | 65,625 | | 57,719 | 47,875 | 67,804 | |
| **M** | **80-84** | 38,439 | 31,420 | 46,228 | 48,734 | | 34,596 | 47,129 | | 48,028 | 36,776 | 47,632 | | 46,973 | 37,689 | 48,874 | 46,267 | | 38,250 | 51,141 | | 45,528 | 38,339 | 53,471 | |
| **M** | **85-89** | 19,220 | 15,384 | 22,901 | 24,648 | | 17,216 | 23,535 | | 25,180 | 19,134 | 24,833 | | 25,885 | 20,585 | 26,573 | 26,463 | | 21,894 | 28,752 | | 26,504 | 22,359 | 30,931 | |
| **M** | **90-94** | 6,030 | 4,950 | 7,112 | 8,151 | | 5,764 | 7,776 | | 8,507 | 6,477 | 8,393 | | 8,685 | 6,894 | 8,905 | 8,804 | | 7,168 | 9,607 | | 8,891 | 7,343 | 10,376 | |
| **M** | **95+** | 729 | 603 | 860 | 965 | | 686 | 919 | | 1,147 | 869 | 1,119 | | 1,364 | 1,084 | 1,390 | 1,542 | | 1,269 | 1,675 | | 1,635 | 1,367 | 1,903 | |
| **M** | **Total** | 532,290 | 496,749 | 569,455 | 631,483 | | 527,895 | 590,008 | | 639,084 | 555,880 | 611,621 | | 646,077 | 579,700 | 637,115 | 652,933 | | 601,680 | 668,543 | | 652,997 | 614,633 | 695,362 | |
| **M** | **Total per 100,000** | 9,747 | 9,096 | 10,428 | 11,503 | | 9,616 | 10,748 | | 11,575 | 10,068 | 11,077 | | 11,635 | 10,440 | 11,474 | 11,695 | | 10,777 | 11,975 | | 11,744 | 11,054 | 12,506 | |

Appendix Table 6: YLDs for OST per sex, age and year

|  |  | **2013** | | | **2014** | | | **2015** | | | **2016** | | | **2017** | | | **2018** | | |
| --- | --- | --- | --- | --- | --- | --- | --- | --- | --- | --- | --- | --- | --- | --- | --- | --- | --- | --- | --- |
| **Sex** | **Age** | **YLD** | **2.5CI** | **97.5CI** | **YLD** | **2.5CI** | **97.5CI** | **YLD** | **2.5CI** | **97.5CI** | **YLD** | **2.5CI** | **97.5CI** | **YLD** | **2.5CI** | **97.5CI** | **YLD** | **2.5CI** | **97.5CI** |
| **F** | **<1** | - | - | - | - | - | - | - | - | - | - | - | - | - | - | - | - | - | - |
| **F** | **1-4** | - | - | - | - | - | - | - | - | - | - | - | - | - | - | - | - | - | - |
| **F** | **5-9** | - | - | - | - | - | - | - | - | - | - | - | - | - | - | - | - | - | - |
| **F** | **10-14** | - | - | - | - | - | - | - | - | - | - | - | - | - | - | - | - | - | - |
| **F** | **15-19** | - | - | - | 26 | 1 | 86 | 52 | 2 | 172 | 79 | 3 | 258 | 105 | 5 | 343 | 131 | 6 | 429 |
| **F** | **20-24** | 143 | 18 | 407 | 177 | 53 | 388 | 209 | 72 | 420 | 239 | 81 | 478 | 269 | 83 | 554 | 302 | 74 | 673 |
| **F** | **25-29** | 387 | 102 | 852 | 394 | 146 | 788 | 402 | 170 | 746 | 411 | 175 | 757 | 418 | 164 | 791 | 420 | 138 | 882 |
| **F** | **30-34** | 597 | 217 | 1,209 | 562 | 245 | 1,064 | 524 | 251 | 951 | 487 | 233 | 892 | 452 | 203 | 865 | 416 | 141 | 920 |
| **F** | **35-39** | 276 | 122 | 510 | 446 | 256 | 688 | 625 | 372 | 954 | 809 | 473 | 1,237 | 993 | 574 | 1,547 | 1,170 | 656 | 1,840 |
| **F** | **40-44** | 1,427 | 833 | 2,113 | 1,368 | 860 | 2,021 | 1,305 | 815 | 2,002 | 1,242 | 731 | 1,960 | 1,187 | 620 | 2,009 | 1,147 | 529 | 2,096 |
| **F** | **45-49** | 2,310 | 1,476 | 3,519 | 2,387 | 1,591 | 3,558 | 2,469 | 1,660 | 3,641 | 2,565 | 1,716 | 3,706 | 2,667 | 1,729 | 3,877 | 2,776 | 1,743 | 4,069 |
| **F** | **50-54** | 3,101 | 2,006 | 4,440 | 3,173 | 2,130 | 4,493 | 3,235 | 2,206 | 4,511 | 3,277 | 2,249 | 4,561 | 3,301 | 2,252 | 4,696 | 3,345 | 2,215 | 4,786 |
| **F** | **55-59** | 3,784 | 2,553 | 5,355 | 4,002 | 2,732 | 5,562 | 4,215 | 2,923 | 5,814 | 4,422 | 3,047 | 6,143 | 4,630 | 3,174 | 6,441 | 4,787 | 3,279 | 6,724 |
| **F** | **60-64** | 4,535 | 3,136 | 6,176 | 4,573 | 3,166 | 6,216 | 4,639 | 3,196 | 6,281 | 4,721 | 3,220 | 6,467 | 4,801 | 3,247 | 6,612 | 4,799 | 3,215 | 6,665 |
| **F** | **65-69** | 4,848 | 3,272 | 6,555 | 5,077 | 3,502 | 6,798 | 5,281 | 3,734 | 7,067 | 5,414 | 3,834 | 7,281 | 5,488 | 3,809 | 7,366 | 5,560 | 3,856 | 7,591 |
| **F** | **70-74** | 3,997 | 2,712 | 5,535 | 3,964 | 2,735 | 5,381 | 3,998 | 2,807 | 5,415 | 4,186 | 2,904 | 5,665 | 4,451 | 3,101 | 6,018 | 4,411 | 3,057 | 5,988 |
| **F** | **75-79** | 3,969 | 2,682 | 5,537 | 3,942 | 2,682 | 5,453 | 3,880 | 2,667 | 5,258 | 3,748 | 2,576 | 5,061 | 3,604 | 2,450 | 4,829 | 3,572 | 2,411 | 4,857 |
| **F** | **80-84** | 3,850 | 2,645 | 5,300 | 3,861 | 2,665 | 5,248 | 3,836 | 2,663 | 5,184 | 3,786 | 2,624 | 5,094 | 3,751 | 2,601 | 5,051 | 3,762 | 2,559 | 5,130 |
| **F** | **85-89** | 2,786 | 1,924 | 3,860 | 2,687 | 1,860 | 3,697 | 2,608 | 1,837 | 3,608 | 2,535 | 1,764 | 3,486 | 2,452 | 1,690 | 3,391 | 2,309 | 1,560 | 3,251 |
| **F** | **90-94** | 1,183 | 764 | 1,682 | 1,293 | 863 | 1,802 | 1,359 | 917 | 1,875 | 1,402 | 944 | 1,921 | 1,448 | 961 | 1,994 | 1,464 | 950 | 2,066 |
| **F** | **95+** | 345 | 227 | 476 | 321 | 217 | 442 | 331 | 221 | 457 | 343 | 224 | 491 | 341 | 198 | 518 | 299 | 138 | 494 |
| **F** | **Total** | 37,539 | 26,223 | 49,912 | 38,252 | 26,666 | 50,782 | 38,971 | 27,178 | 51,837 | 39,666 | 27,547 | 52,792 | 40,358 | 28,143 | 53,958 | 40,670 | 28,333 | 54,750 |
| **F** | **Total per 100,000** | 663 | 463 | 881 | 672 | 469 | 892 | 682 | 475 | 907 | 691 | 480 | 919 | 700 | 488 | 936 | 708 | 494 | 954 |
| **F** | **Age-std per 100,000** | 646 |  |  | 652 |  |  | 658 |  |  | 664 |  |  | 671 |  |  | 677 |  |  |
| **M** | **<1** | - | - | - | - | - | - | - | - | - | - | - | - | - | - | - | - | - | - |
| **M** | **1-4** | - | - | - | - | - | - | - | - | - | - | - | - | - | - | - | - | - | - |
| **M** | **5-9** | - | - | - | - | - | - | - | - | - | - | - | - | - | - | - | - | - | - |
| **M** | **10-14** | - | - | - | - | - | - | - | - | - | - | - | - | - | - | - | - | - | - |
| **M** | **15-19** | - | - | - | - | - | - | - | - | - | - | - | - | - | - | - | - | - | - |
| **M** | **20-24** | - | - | - | 17 | - | 61 | 34 | 1 | 121 | 50 | 1 | 179 | 66 | 2 | 237 | 82 | 2 | 296 |
| **M** | **25-29** | 203 | 51 | 457 | 177 | 50 | 381 | 151 | 48 | 315 | 125 | 42 | 258 | 97 | 31 | 216 | 67 | 7 | 197 |
| **M** | **30-34** | 473 | 132 | 1,028 | 420 | 140 | 865 | 367 | 143 | 717 | 315 | 131 | 596 | 264 | 109 | 511 | 214 | 52 | 505 |
| **M** | **35-39** | 480 | 166 | 958 | 564 | 265 | 1,003 | 655 | 333 | 1,092 | 748 | 373 | 1,266 | 840 | 404 | 1,460 | 926 | 416 | 1,696 |
| **M** | **40-44** | 901 | 441 | 1,506 | 954 | 521 | 1,469 | 1,001 | 585 | 1,502 | 1,044 | 628 | 1,581 | 1,091 | 643 | 1,677 | 1,152 | 635 | 1,835 |
| **M** | **45-49** | 1,461 | 871 | 2,273 | 1,477 | 915 | 2,209 | 1,497 | 960 | 2,175 | 1,523 | 969 | 2,229 | 1,551 | 970 | 2,293 | 1,587 | 922 | 2,453 |
| **M** | **50-54** | 1,940 | 1,202 | 2,909 | 2,080 | 1,338 | 2,989 | 2,218 | 1,452 | 3,148 | 2,344 | 1,556 | 3,416 | 2,458 | 1,601 | 3,686 | 2,577 | 1,596 | 3,964 |
| **M** | **55-59** | 2,802 | 1,828 | 4,076 | 2,988 | 2,007 | 4,281 | 3,177 | 2,197 | 4,421 | 3,364 | 2,303 | 4,641 | 3,551 | 2,464 | 4,968 | 3,693 | 2,530 | 5,260 |
| **M** | **60-64** | 2,592 | 1,675 | 3,694 | 2,655 | 1,779 | 3,734 | 2,732 | 1,862 | 3,771 | 2,821 | 1,902 | 3,866 | 2,914 | 1,963 | 3,997 | 2,957 | 1,964 | 4,163 |
| **M** | **65-69** | 2,414 | 1,579 | 3,448 | 2,620 | 1,774 | 3,605 | 2,821 | 1,945 | 3,805 | 2,981 | 2,041 | 4,064 | 3,109 | 2,107 | 4,226 | 3,233 | 2,144 | 4,402 |
| **M** | **70-74** | 2,242 | 1,452 | 3,221 | 2,260 | 1,500 | 3,222 | 2,312 | 1,564 | 3,255 | 2,449 | 1,685 | 3,373 | 2,635 | 1,801 | 3,628 | 2,627 | 1,726 | 3,690 |
| **M** | **75-79** | 1,765 | 1,135 | 2,551 | 1,835 | 1,232 | 2,574 | 1,890 | 1,279 | 2,612 | 1,908 | 1,288 | 2,653 | 1,915 | 1,269 | 2,667 | 1,967 | 1,278 | 2,759 |
| **M** | **80-84** | 1,398 | 886 | 2,078 | 1,554 | 1,032 | 2,216 | 1,696 | 1,139 | 2,340 | 1,826 | 1,243 | 2,529 | 1,969 | 1,346 | 2,765 | 2,101 | 1,388 | 2,961 |
| **M** | **85-89** | 916 | 536 | 1,423 | 896 | 548 | 1,349 | 885 | 541 | 1,330 | 877 | 536 | 1,311 | 861 | 493 | 1,306 | 806 | 417 | 1,300 |
| **M** | **90-94** | 309 | 124 | 540 | 309 | 142 | 514 | 298 | 157 | 480 | 278 | 160 | 431 | 253 | 140 | 396 | 219 | 103 | 378 |
| **M** | **95+** | - | - | - | 7 | - | 17 | 18 | 1 | 42 | 33 | 1 | 76 | 50 | 2 | 117 | 63 | 3 | 146 |
| **M** | **Total** | 19,896 | 13,646 | 26,676 | 20,814 | 14,394 | 27,652 | 21,750 | 15,138 | 28,908 | 22,686 | 15,863 | 29,939 | 23,624 | 16,613 | 30,925 | 24,272 | 17,038 | 32,156 |
| **M** | **Total per 100,000** | 364 | 250 | 488 | 379 | 262 | 504 | 394 | 274 | 524 | 409 | 286 | 539 | 423 | 298 | 554 | 438 | 307 | 580 |
| **M** | **Age-std per 100,000** | 401 |  |  | 413 |  |  | 424 |  |  | 436 |  |  | 447 |  |  | 459 |  |  |

Appendix Table 7: Number of RHE cases by sex, age and year

|  |  | **2013** | | | **2014** | | | **2015** | | | | **2016** | | | | **2017** | | | | **2018** | | | |  |
| --- | --- | --- | --- | --- | --- | --- | --- | --- | --- | --- | --- | --- | --- | --- | --- | --- | --- | --- | --- | --- | --- | --- | --- | --- |
| **Sex** | **Age** | **N** | **2.5CI** | **97.5CI** | **N** | **2.5CI** | **97.5CI** | | **N** | **2.5CI** | **97.5CI** | | **N** | **2.5CI** | **97.5CI** | | **N** | **2.5CI** | **97.5CI** | | **N** | **2.5CI** | **97.5CI** | |
| **F** | **<1** | - | - | - | - | - | - | | - | - | - | | - | - | - | | - | - | - | | - | - | - | |
| **F** | **1-4** | - | - | - | - | - | - | | - | - | - | | - | - | - | | - | - | - | | - | - | - | |
| **F** | **5-9** | - | - | - | 8 | 2 | 18 | | 45 | 10 | 121 | | 77 | 25 | 186 | | 75 | 25 | 159 | | 128 | 57 | 230 | |
| **F** | **10-14** | 77 | 20 | 160 | 105 | 38 | 208 | | 84 | 31 | 168 | | 159 | 67 | 288 | | 175 | 82 | 285 | | 151 | 69 | 267 | |
| **F** | **15-19** | 217 | 100 | 389 | 123 | 62 | 210 | | 161 | 92 | 251 | | 269 | 160 | 398 | | 347 | 224 | 496 | | 494 | 327 | 674 | |
| **F** | **20-24** | 657 | 448 | 912 | 839 | 596 | 1,119 | | 623 | 432 | 851 | | 460 | 309 | 658 | | 393 | 284 | 532 | | 389 | 259 | 548 | |
| **F** | **25-29** | 975 | 702 | 1,281 | 813 | 604 | 1,048 | | 745 | 563 | 977 | | 757 | 577 | 986 | | 847 | 677 | 1,054 | | 780 | 592 | 1,004 | |
| **F** | **30-34** | 929 | 652 | 1,225 | 1,154 | 881 | 1,463 | | 1,307 | 1,047 | 1,619 | | 1,508 | 1,219 | 1,866 | | 1,429 | 1,205 | 1,676 | | 1,681 | 1,402 | 1,991 | |
| **F** | **35-39** | 2,075 | 1,634 | 2,508 | 2,002 | 1,610 | 2,444 | | 1,589 | 1,267 | 1,914 | | 1,956 | 1,633 | 2,313 | | 1,703 | 1,458 | 1,984 | | 2,003 | 1,669 | 2,341 | |
| **F** | **40-44** | 2,875 | 2,348 | 3,423 | 3,194 | 2,704 | 3,772 | | 2,876 | 2,451 | 3,342 | | 2,687 | 2,260 | 3,149 | | 2,194 | 1,893 | 2,519 | | 2,538 | 2,176 | 2,934 | |
| **F** | **45-49** | 2,862 | 2,318 | 3,424 | 2,919 | 2,449 | 3,405 | | 3,142 | 2,691 | 3,647 | | 3,481 | 3,011 | 4,014 | | 2,779 | 2,444 | 3,156 | | 3,542 | 3,069 | 4,087 | |
| **F** | **50-54** | 4,359 | 3,755 | 5,019 | 4,357 | 3,773 | 4,949 | | 4,190 | 3,668 | 4,715 | | 4,156 | 3,619 | 4,700 | | 3,519 | 3,131 | 3,981 | | 4,098 | 3,568 | 4,635 | |
| **F** | **55-59** | 4,478 | 3,889 | 5,187 | 4,325 | 3,744 | 4,961 | | 4,237 | 3,654 | 4,822 | | 4,442 | 3,931 | 5,017 | | 4,373 | 3,891 | 4,890 | | 4,465 | 3,970 | 5,028 | |
| **F** | **60-64** | 6,091 | 5,320 | 6,873 | 5,808 | 5,086 | 6,550 | | 5,728 | 5,118 | 6,390 | | 5,363 | 4,780 | 5,970 | | 4,362 | 3,915 | 4,874 | | 5,115 | 4,586 | 5,744 | |
| **F** | **65-69** | 5,063 | 4,412 | 5,855 | 5,567 | 4,885 | 6,314 | | 5,530 | 4,887 | 6,176 | | 5,901 | 5,257 | 6,558 | | 5,274 | 4,763 | 5,787 | | 5,933 | 5,319 | 6,523 | |
| **F** | **70-74** | 4,011 | 3,403 | 4,696 | 4,035 | 3,435 | 4,677 | | 4,035 | 3,498 | 4,658 | | 4,247 | 3,702 | 4,844 | | 4,377 | 3,893 | 4,920 | | 4,835 | 4,236 | 5,421 | |
| **F** | **75-79** | 5,679 | 4,940 | 6,484 | 5,110 | 4,469 | 5,821 | | 4,696 | 4,085 | 5,357 | | 4,462 | 3,924 | 5,085 | | 3,696 | 3,271 | 4,141 | | 3,869 | 3,360 | 4,444 | |
| **F** | **80-84** | 3,098 | 2,513 | 3,697 | 3,282 | 2,739 | 3,876 | | 3,453 | 2,938 | 4,039 | | 3,487 | 2,976 | 4,057 | | 3,897 | 3,417 | 4,387 | | 4,132 | 3,616 | 4,681 | |
| **F** | **85-89** | 2,163 | 1,693 | 2,723 | 2,162 | 1,663 | 2,648 | | 1,962 | 1,584 | 2,400 | | 1,952 | 1,595 | 2,350 | | 2,050 | 1,732 | 2,425 | | 2,080 | 1,735 | 2,467 | |
| **F** | **90-94** | 1,060 | 712 | 1,525 | 1,051 | 678 | 1,498 | | 1,171 | 862 | 1,573 | | 1,042 | 759 | 1,382 | | 1,173 | 967 | 1,409 | | 893 | 708 | 1,121 | |
| **F** | **95+** | 109 | 15 | 319 | 174 | 42 | 371 | | 155 | 46 | 246 | | 240 | 94 | 322 | | 477 | 190 | 308 | | 703 | 274 | 464 | |
| **F** | **Total** | 46,779 | 44,764 | 49,153 | 47,028 | 45,005 | 49,024 | | 45,730 | 43,856 | 47,679 | | 46,643 | 44,944 | 48,399 | | 43,140 | 41,516 | 44,457 | | 47,828 | 45,749 | 49,276 | |
| **F** | **Total per 100,000** | 826 | 790 | 868 | 826 | 791 | 862 | | 800 | 767 | 834 | | 812 | 783 | 843 | | 748 | 720 | 771 | | 834 | 798 | 859 | |
| **M** | **<1** | - | - | - | - | - | - | | - | - | - | | - | - | - | | - | - | - | | - | - | - | |
| **M** | **1-4** | - | - | - | - | - | - | | - | - | - | | - | - | - | | - | - | - | | - | - | - | |
| **M** | **5-9** | 51 | 7 | 146 | 16 | - | 63 | | 12 | - | 48 | | 8 | - | 32 | | 4 | - | 16 | | - | - | - | |
| **M** | **10-14** | 102 | 31 | 227 | 132 | 45 | 279 | | 68 | 22 | 157 | | 146 | 62 | 281 | | 119 | 54 | 220 | | 126 | 53 | 235 | |
| **M** | **15-19** | 214 | 92 | 380 | 208 | 86 | 372 | | 340 | 182 | 550 | | 297 | 157 | 467 | | 240 | 137 | 391 | | 228 | 124 | 366 | |
| **M** | **20-24** | 359 | 180 | 571 | 347 | 181 | 557 | | 233 | 132 | 376 | | 453 | 277 | 677 | | 433 | 280 | 601 | | 458 | 294 | 664 | |
| **M** | **25-29** | 670 | 414 | 958 | 729 | 495 | 1,021 | | 641 | 429 | 907 | | 579 | 408 | 829 | | 523 | 381 | 684 | | 654 | 473 | 863 | |
| **M** | **30-34** | 1,074 | 764 | 1,424 | 1,180 | 863 | 1,545 | | 1,069 | 788 | 1,405 | | 1,231 | 953 | 1,557 | | 932 | 722 | 1,143 | | 934 | 704 | 1,193 | |
| **M** | **35-39** | 1,365 | 1,022 | 1,777 | 1,352 | 1,014 | 1,723 | | 1,470 | 1,173 | 1,845 | | 1,314 | 1,017 | 1,647 | | 1,367 | 1,107 | 1,661 | | 1,446 | 1,139 | 1,763 | |
| **M** | **40-44** | 2,528 | 2,021 | 3,088 | 2,322 | 1,833 | 2,847 | | 2,055 | 1,669 | 2,478 | | 1,919 | 1,558 | 2,349 | | 1,596 | 1,333 | 1,870 | | 1,674 | 1,366 | 2,022 | |
| **M** | **45-49** | 1,673 | 1,267 | 2,140 | 1,720 | 1,352 | 2,139 | | 1,676 | 1,338 | 2,079 | | 1,953 | 1,585 | 2,389 | | 1,973 | 1,656 | 2,322 | | 2,387 | 1,969 | 2,811 | |
| **M** | **50-54** | 2,999 | 2,464 | 3,610 | 2,709 | 2,250 | 3,191 | | 2,660 | 2,216 | 3,160 | | 2,565 | 2,128 | 3,027 | | 2,287 | 1,970 | 2,668 | | 2,447 | 2,050 | 2,868 | |
| **M** | **55-59** | 3,305 | 2,763 | 3,900 | 3,521 | 3,000 | 4,106 | | 3,355 | 2,843 | 3,900 | | 3,281 | 2,793 | 3,769 | | 3,060 | 2,665 | 3,451 | | 3,587 | 3,112 | 4,111 | |
| **M** | **60-64** | 2,477 | 1,983 | 3,009 | 2,518 | 2,067 | 3,014 | | 2,664 | 2,246 | 3,153 | | 2,967 | 2,513 | 3,465 | | 2,712 | 2,348 | 3,124 | | 3,396 | 2,904 | 3,925 | |
| **M** | **65-69** | 2,731 | 2,223 | 3,315 | 2,814 | 2,330 | 3,344 | | 2,673 | 2,210 | 3,186 | | 2,828 | 2,379 | 3,317 | | 2,706 | 2,320 | 3,152 | | 3,024 | 2,589 | 3,492 | |
| **M** | **70-74** | 2,606 | 2,137 | 3,162 | 2,684 | 2,187 | 3,217 | | 2,622 | 2,166 | 3,105 | | 2,824 | 2,382 | 3,303 | | 2,526 | 2,150 | 2,911 | | 2,788 | 2,361 | 3,249 | |
| **M** | **75-79** | 1,969 | 1,552 | 2,478 | 2,228 | 1,761 | 2,684 | | 2,187 | 1,801 | 2,647 | | 2,100 | 1,709 | 2,514 | | 2,239 | 1,888 | 2,647 | | 2,268 | 1,882 | 2,698 | |
| **M** | **80-84** | 1,555 | 1,144 | 2,028 | 1,635 | 1,265 | 2,073 | | 1,721 | 1,334 | 2,163 | | 1,753 | 1,396 | 2,139 | | 1,723 | 1,420 | 2,058 | | 1,792 | 1,445 | 2,136 | |
| **M** | **85-89** | 1,050 | 724 | 1,453 | 942 | 647 | 1,305 | | 884 | 609 | 1,193 | | 876 | 633 | 1,168 | | 998 | 799 | 1,223 | | 1,063 | 822 | 1,346 | |
| **M** | **90-94** | - | - | - | 156 | 45 | 333 | | 157 | 60 | 312 | | 233 | 123 | 390 | | 327 | 238 | 426 | | 313 | 213 | 430 | |
| **M** | **95+** | - | - | - | 5 | 1 | 3 | | 13 | 3 | 8 | | 23 | 5 | 14 | | 109 | 37 | 73 | | 183 | 41 | 105 | |
| **M** | **Total** | 26,728 | 25,082 | 28,495 | 27,219 | 25,539 | 28,905 | | 26,501 | 25,078 | 27,926 | | 27,352 | 25,897 | 28,799 | | 25,873 | 24,570 | 26,985 | | 28,768 | 27,373 | 30,020 | |
| **M** | **Total per 100,000** | 489 | 459 | 522 | 496 | 465 | 527 | | 480 | 454 | 506 | | 493 | 466 | 519 | | 463 | 440 | 483 | | 517 | 492 | 540 | |

Appendix Table 8: YLD for RHE and confidence intervals by sex, age and year

|  |  | **2013** | | | **2014** | | | **2015** | | | **2016** | | | **2017** | | | **2018** | | |
| --- | --- | --- | --- | --- | --- | --- | --- | --- | --- | --- | --- | --- | --- | --- | --- | --- | --- | --- | --- |
| **Sex** | **Age** | **YLD** | **2.5CI** | **97.5CI** | **YLD** | **2.5CI** | **97.5CI** | **YLD** | **2.5CI** | **97.5CI** | **YLD** | **2.5CI** | **97.5CI** | **YLD** | **2.5CI** | **97.5CI** | **YLD** | **2.5CI** | **97.5CI** |
| **F** | **<1** | - | - | - | - | - | - | - | - | - | - | - | - | - | - | - | - | - | - |
| **F** | **1-4** | - | - | - | - | - | - | - | - | - | - | - | - | - | - | - | - | - | - |
| **F** | **5-9** | - | - | - | - | - | - | - | - | - | - | - | - | - | - | - | - | - | - |
| **F** | **10-14** | - | - | - | - | - | - | - | - | - | - | - | - | - | - | - | - | - | - |
| **F** | **15-19** | 40 | 5 | 107 | 57 | 24 | 116 | 75 | 38 | 132 | 93 | 50 | 155 | 111 | 59 | 184 | 128 | 67 | 217 |
| **F** | **20-24** | 261 | 140 | 424 | 230 | 130 | 362 | 198 | 119 | 305 | 166 | 103 | 249 | 134 | 82 | 208 | 105 | 53 | 181 |
| **F** | **25-29** | 343 | 199 | 536 | 319 | 195 | 481 | 297 | 189 | 434 | 273 | 185 | 386 | 248 | 165 | 350 | 220 | 141 | 328 |
| **F** | **30-34** | 206 | 103 | 348 | 264 | 168 | 397 | 322 | 222 | 451 | 379 | 267 | 520 | 437 | 309 | 603 | 495 | 346 | 682 |
| **F** | **35-39** | 609 | 394 | 894 | 611 | 413 | 863 | 618 | 436 | 850 | 626 | 460 | 840 | 631 | 461 | 838 | 629 | 447 | 855 |
| **F** | **40-44** | 814 | 533 | 1,189 | 800 | 558 | 1,128 | 782 | 565 | 1,061 | 764 | 557 | 1,021 | 750 | 548 | 996 | 746 | 533 | 1,005 |
| **F** | **45-49** | 886 | 609 | 1,261 | 916 | 669 | 1,250 | 947 | 715 | 1,266 | 984 | 742 | 1,298 | 1,024 | 766 | 1,364 | 1,065 | 774 | 1,429 |
| **F** | **50-54** | 1,441 | 1,024 | 1,981 | 1,384 | 1,005 | 1,859 | 1,320 | 976 | 1,747 | 1,248 | 924 | 1,620 | 1,170 | 873 | 1,536 | 1,099 | 804 | 1,460 |
| **F** | **55-59** | 1,475 | 1,037 | 2,002 | 1,455 | 1,048 | 1,943 | 1,429 | 1,056 | 1,890 | 1,399 | 1,050 | 1,822 | 1,366 | 1,021 | 1,780 | 1,318 | 967 | 1,731 |
| **F** | **60-64** | 1,868 | 1,311 | 2,531 | 1,743 | 1,262 | 2,321 | 1,625 | 1,197 | 2,142 | 1,508 | 1,124 | 1,976 | 1,385 | 1,032 | 1,812 | 1,236 | 902 | 1,639 |
| **F** | **65-69** | 1,550 | 1,084 | 2,050 | 1,586 | 1,153 | 2,070 | 1,613 | 1,194 | 2,080 | 1,616 | 1,217 | 2,094 | 1,601 | 1,212 | 2,084 | 1,585 | 1,183 | 2,063 |
| **F** | **70-74** | 1,416 | 1,009 | 1,883 | 1,330 | 963 | 1,737 | 1,265 | 930 | 1,642 | 1,243 | 921 | 1,599 | 1,233 | 905 | 1,587 | 1,134 | 812 | 1,493 |
| **F** | **75-79** | 1,670 | 1,203 | 2,220 | 1,564 | 1,145 | 2,052 | 1,444 | 1,076 | 1,878 | 1,302 | 973 | 1,679 | 1,161 | 860 | 1,502 | 1,058 | 760 | 1,398 |
| **F** | **80-84** | 1,057 | 740 | 1,483 | 1,072 | 781 | 1,450 | 1,076 | 807 | 1,408 | 1,073 | 821 | 1,395 | 1,074 | 821 | 1,390 | 1,088 | 822 | 1,422 |
| **F** | **85-89** | 619 | 411 | 885 | 603 | 417 | 838 | 593 | 427 | 801 | 583 | 428 | 774 | 572 | 413 | 772 | 547 | 385 | 762 |
| **F** | **90-94** | 195 | 105 | 324 | 205 | 124 | 321 | 208 | 132 | 308 | 207 | 131 | 297 | 205 | 129 | 300 | 200 | 115 | 307 |
| **F** | **95+** | 42 | 16 | 84 | 50 | 26 | 88 | 65 | 37 | 106 | 84 | 48 | 133 | 104 | 56 | 167 | 115 | 57 | 190 |
| **F** | **Total** | 14,494 | 11,139 | 18,391 | 14,189 | 11,028 | 17,887 | 13,876 | 10,816 | 17,508 | 13,547 | 10,608 | 17,066 | 13,206 | 10,351 | 16,683 | 12,768 | 9,982 | 16,090 |
| **F** | **Total per 100,000** | 267 | 209 | 336 | 261 | 206 | 326 | 256 | 203 | 319 | 252 | 201 | 314 | 241 | 191 | 300 | 237 | 188 | 295 |
| **F** | **Age-std per 100,000** | 263 |  |  | 255 |  |  | 249 |  |  | 245 |  |  | 233 |  |  | 228 |  |  |
| **M** | **<1** | - | - | - | - | - | - | - | - | - | - | - | - | - | - | - | - | - | - |
| **M** | **1-4** | - | - | - | - | - | - | - | - | - | - | - | - | - | - | - | - | - | - |
| **M** | **5-9** | - | - | - | - | - | - | - | - | - | - | - | - | - | - | - | - | - | - |
| **M** | **10-14** | - | - | - | - | - | - | - | - | - | - | - | - | - | - | - | - | - | - |
| **M** | **15-19** | 86 | 22 | 185 | 77 | 26 | 158 | 68 | 27 | 134 | 60 | 25 | 111 | 52 | 21 | 99 | 43 | 13 | 98 |
| **M** | **20-24** | 193 | 91 | 352 | 187 | 100 | 312 | 181 | 106 | 286 | 174 | 106 | 263 | 168 | 96 | 255 | 163 | 83 | 265 |
| **M** | **25-29** | 126 | 47 | 239 | 137 | 69 | 238 | 149 | 86 | 235 | 162 | 97 | 246 | 174 | 105 | 263 | 184 | 106 | 286 |
| **M** | **30-34** | 288 | 157 | 474 | 280 | 166 | 434 | 272 | 173 | 397 | 265 | 174 | 379 | 258 | 167 | 379 | 252 | 152 | 390 |
| **M** | **35-39** | 480 | 283 | 728 | 474 | 310 | 688 | 472 | 325 | 664 | 470 | 333 | 648 | 466 | 330 | 633 | 459 | 314 | 635 |
| **M** | **40-44** | 652 | 414 | 976 | 633 | 421 | 917 | 612 | 429 | 848 | 590 | 418 | 806 | 572 | 407 | 793 | 563 | 379 | 804 |
| **M** | **45-49** | 467 | 268 | 709 | 502 | 327 | 717 | 538 | 379 | 744 | 576 | 415 | 782 | 614 | 435 | 826 | 655 | 455 | 893 |
| **M** | **50-54** | 969 | 651 | 1,346 | 926 | 646 | 1,257 | 879 | 634 | 1,188 | 827 | 609 | 1,088 | 771 | 561 | 1,027 | 716 | 504 | 980 |
| **M** | **55-59** | 753 | 484 | 1,095 | 816 | 572 | 1,132 | 880 | 641 | 1,170 | 945 | 697 | 1,251 | 1,010 | 733 | 1,349 | 1,062 | 759 | 1,437 |
| **M** | **60-64** | 800 | 513 | 1,150 | 816 | 567 | 1,128 | 836 | 605 | 1,117 | 859 | 631 | 1,142 | 884 | 652 | 1,187 | 893 | 648 | 1,221 |
| **M** | **65-69** | 766 | 507 | 1,097 | 790 | 551 | 1,090 | 811 | 579 | 1,078 | 819 | 598 | 1,072 | 818 | 596 | 1,072 | 815 | 588 | 1,092 |
| **M** | **70-74** | 972 | 673 | 1,344 | 907 | 655 | 1,228 | 853 | 616 | 1,125 | 824 | 600 | 1,075 | 800 | 586 | 1,051 | 711 | 500 | 971 |
| **M** | **75-79** | 656 | 434 | 928 | 669 | 464 | 909 | 676 | 484 | 895 | 670 | 496 | 892 | 661 | 480 | 894 | 668 | 473 | 926 |
| **M** | **80-84** | 585 | 363 | 876 | 571 | 379 | 821 | 550 | 383 | 760 | 524 | 372 | 707 | 501 | 350 | 671 | 475 | 319 | 658 |
| **M** | **85-89** | 355 | 216 | 542 | 340 | 218 | 497 | 327 | 219 | 464 | 315 | 215 | 436 | 299 | 200 | 421 | 268 | 171 | 393 |
| **M** | **90-94** | 33 | 6 | 82 | 39 | 13 | 81 | 46 | 21 | 80 | 51 | 25 | 88 | 57 | 28 | 104 | 61 | 25 | 116 |
| **M** | **95+** | - | - | - | 1 | - | 3 | 2 | - | 8 | 4 | - | 15 | 6 | - | 23 | 8 | - | 29 |
| **M** | **Total** | 8,180 | 6,232 | 10,415 | 8,166 | 6,340 | 10,367 | 8,153 | 6,365 | 10,266 | 8,136 | 6,347 | 10,236 | 8,110 | 6,328 | 10,192 | 7,996 | 6,233 | 10,029 |
| **M** | **Total per 100,000** | 155 | 121 | 197 | 153 | 119 | 193 | 154 | 122 | 193 | 152 | 120 | 190 | 150 | 118 | 188 | 148 | 117 | 185 |
| **M** | **Age-std per 100,000** | 168 |  |  | 164 |  |  | 164 |  |  | 161 |  |  | 157 |  |  | 154 |  |  |

Appendix Table 9: Sociodemographic characteristics of participants to HIS 2013 - at the time of the survey

|  | HIS 2013 sample –  adult population with complete cost data | | HIS 2013 sample – working population | |
| --- | --- | --- | --- | --- |
|  | N^(1)^ | %^(2)^ | N | % |
| **Total** | 9,814 | 100% | 3,941 |  |
| **Gender** |  |  |  |  |
| Men | 4,719 | 48% | 2,049 | 52% |
| Women | 5,095 | 52% | 1,892 | 48% |
| **Age (mean)** | 42.5 | - | 42.3 | - |
| **Education** |  |  |  |  |
| No diploma/primary | 1,038 | 8% | 152 | 2% |
| Lower secondary | 1,368 | 12% | 367 | 7% |
| Higher secondary | 3,145 | 33% | 1,294 | 32% |
| Higher education | 4,113 | 48% | 2,102 | 59% |
| **Household highest income level** | |  |  |  |
| Quintile 1 | 1,943 | 12% | 378 | 6% |
| Quintile 2 | 1,498 | 15% | 420 | 8% |
| Quintile 3 | 1,721 | 21% | 751 | 21% |
| Quintile 4 | 1,736 | 25% | 859 | 28% |
| Quintile 5 | 1,744 | 28% | 1,076 | 36% |
| **Nationality** |  |  |  |  |
| Belgian | 8,736 | 95% | 3,464 | 94% |
| Non-Belgian | 640 | 3% | 324 | 4% |
| Non-Belgian Non-EU | 431 | 2% | 152 | 2% |

1. Number of participants in the survey
2. Survey weighted prevalence

Appendix Table 10: Unadjusted direct average annual costs in function of MSK disorders (univariate regression, Belgian adult population)

| **MSK disorders** | **Cost ratio**  **(95% CI)** | **Std. error** | **P-value** | **Mean incremental cost**  **(95% CI)** |
| --- | --- | --- | --- | --- |
| Low back pain | 2.41  (2.10 – 2.77) | 0.17 | <0.001 | 4,614  (3,677 – 5,545) |
| Intercept | 3,291 | 120 | <0.001 | - |
| Neck pain | 2.33  (1.98 – 2.75) | 0.20 | <0.001 | 4,625  (3,493 – 5,932) |
| Intercept | 3,480 | 120 | <0.001 | - |
| Osteoarthritis | 2.45  (2.16 – 2.78) | 0.16 | <0.001 | 4,397  (3,639 – 5,158) |
| Intercept | 3,032 | 117 | <0.001 | - |
| Rheumatoid arthritis | 2.31  (1.98 – 2.71) | 0.19 | <0.001 | 4,496  (3,462 – 5,646) |
| Intercept | 3,418 | 119 | <0.001 | - |

Appendix Table 11: Unadjusted indirect average annual costs in function of MSK disorders (univariate regression, Belgian working population)

| **MSK disorders** | **Cost ratio**  **(95% CI)** | **Std. error** | **P-value** | **Mean incremental cost**  **(95% CI)** |
| --- | --- | --- | --- | --- |
| Low back pain | 2.56  (1.79 – 3.66) | 0.47 | <0.001 | 4,336  (2,130 – 6,957) |
| Intercept | 2,751 | 224 | <0.001 | - |
| Neck pain | 3.50  (2.30 – 5.34) | 0.75 | <0.001 | 6,968  (3,589 – 10,695) |
| Intercept | 2,788 | 221 | <0.001 | - |
| Osteoarthritis | 2.37  (1.69 – 3.33) | 0.41 | <0.001 | 3,720  (1,876 – 5,702) |
| Intercept | 2,703 | 227 | <0.001 | - |
| Rheumatoid arthritis | 2.68  (1.71 – 4.20) | 0.61 | <0.001 | 4,719  (1,917 – 8,166) |
| Intercept | 2,814 | 220 | <0.001 | - |

Appendix Table 12: Results of the double selection process (coefficients and standard errors). The cost models include the variables that were significant either in the disease or the cost model, as described in the methods section.

|  | **LBP** | **NKP** | **OST** | **RHE** | **Average direct cost** | **Average indirect cost** | |
| --- | --- | --- | --- | --- | --- | --- | --- |
| **MSK disorder (Ref: No)** |  |  |  |  |  |  | |
| Low back pain | - | - | - | - | 0.360** (0.113) | 0.967** (0.362) | |
| Neck pain | - | - | - | - | 0.309* (0.143) | -0.044 (0.434) | |
| Osteoarthritis | - | - | - | - | 0.049 (0.096) | 0.358 (0.245) | |
| Rheumatoid arthritis | - | - | - | - | 0.067 (0.109) | 0.286 (0.327) | |
| **Sex** |  |  |  |  |  |  | |
| Male | Excluded |  | Ref | Ref | Ref | Ref | |
| Female |  | 0.655*** (0.191) | 0.746*** (0.100) | 0.322* (0.126) | -0.190* (0.082) | 0.592*** (0.167) | |
| **Age** | 0.024*** (0.005) | 0.029*** (0.005) | 0.061*** (0.003) | 0.039*** (0.004) | 0.026*** (0.003) | -0.003 (0.008) | |
| **Education** |  |  |  |  |  |  | |
| Higher education | Ref |  |  |  | Ref |  | |
| Higher secondary | 0.208 (0.184) | Excluded | Excluded | Excluded | -0.035 (0.091) | Excluded | |
| Lower secondary | 0.400* (0.232) |  |  |  | 0.140 (0.111) |  | |
| No diploma or primary education | 0.583* (0.255) |  |  |  | 0.397* (0.155) |  | |
| **At risk due to a lack of leisure time physical activity** | | | | | | |  |
| No | Ref | Ref | Excluded | Excluded | Ref | Ref | |
| Yes | 0.865*** (0.149) | 0.474* (0.193) |  |  | 0.368*** (0.082) | 0.134 (0.215) | |
| **Heavy daily smokers** |  |  |  |  |  |  | |
| No | Ref | Ref | Ref | Ref | Ref | Ref | |
| Yes | 0.966*** (0.215) | 1.056*** (0.268) | 0.628*** (0.190) | 0.496* (0.252) | 0.202 (0.145) | 0.298 (0.253) | |
| **BMI status** |  |  |  |  |  |  | |
| Underweight | 0.689* (0.323) | 0.514 (0.373) | -0.149 (0.311) |  | 0.179 (0.138) | 0.222 (0.493) | |
| Normal weight | Ref | Ref | Ref | Excluded | Ref | Ref | |
| Overweight | 0.431* (0.172) | 0.492* (0.223) | 0.428*** (0.118) |  | 0.032 (0.087) | 0.185 (0.163) | |
| Obese | 1.064*** (0.191) | 0.935*** (0.239) | 0.726*** (0.138) |  | 0.002 (0.111) | 0.534 (0.340) | |

***p<0.0001, **p<0.05, *p<0.10, ‘ ‘ p>0.10
